# Computational prediction of C–H hydricities and their use in predicting the regioselectivity of electron-rich C–H functionalisation reactions

**DOI:** 10.3762/bjoc.22.46

**Published:** 2026-04-17

**Authors:** Rasmus M Borup, Nicolai Ree, Jan H Jensen

**Affiliations:** 1 Department of Chemistry, University of Copenhagen, Copenhagen, DK-2100, Denmark

**Keywords:** bond dissociation energy, hydricity, hydride affinity, hydride-transfer reactions, machine learning (ML), quantum chemistry (QM)

## Abstract

We present HAlator, a fully automated quantum chemistry (QM) workflow for computing C–H hydricities and explore its potential in predicting the regioselectivity of electron-rich C–H functionalisation reactions. The workflow was benchmarked against 35 experimentally determined C–H hydricities in DMSO, yielding a mean absolute error (MAE) of 4.43 kcal/mol and a root mean squared error (RMSE) of 5.45 kcal/mol. Leveraging this approach, we generated a dataset of 3278 C–H sites across 740 molecules to train a machine learning (ML) model based on CM5 atomic charge descriptors, achieving an MAE of 2.30 kcal/mol and an RMSE of 3.74 kcal/mol relative to QM-computed hydricities. The method was further applied to 250 hydride transfer-like reactions, including C–N, C–C, and C–X bond formations, carbene insertions, and oxidative transformations. Comparative analysis with ALFABET, a bond dissociation energy (BDE)-based ML model, reveals that hydricity predictions, when combined with steric accessibility, correctly identify the reactive site in eight out of ten representative reactions, surpassing BDEs in most cases. These findings highlight hydricity as a complementary and, in some cases, superior descriptor for guiding regioselectivity predictions in electron-rich C–H functionalisation. The model is made available at regioselect.org, together with a host of other reactivity predictors.

## Introduction

**Figure 1:**
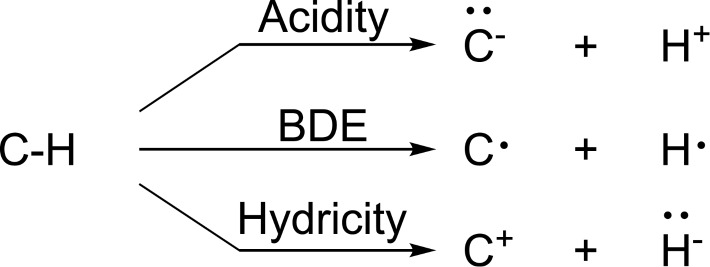
Different cleavage methods for C–H bonds. Heterolytic bond dissociation energy with proton dissociation (acidity, p*K*_a_); homolytic bond dissociation energy (BDE); heterolytic bond dissociation energy with hydride dissociation (hydricity).

Bond dissociation energies (BDEs) and p*K*_a_ values for C–H bonds are often used to rationalise and predict the regioselectivity of various C–H functionalisation reactions and machine learning (ML) models have been developed for both properties [1–5]. In contrast, C–H hydricities have received considerably little attention. However, the insertion into, or H-abstraction from, innately electron-rich C–H bonds are one of the most common C–H functionalisation approaches for sp^3^ C–H bonds [6]. Despite the prevalence of these transformations, predicting which of several electron-rich C–H sites will react remains challenging, especially when electronic and steric effects compete. As Cernak et al. [6] have pointed out, “the reacting C–H bond tends to be electron-rich or adorned with substituents that can stabilise the formation of a developing positive charge.” It is thus possible that predicted hydricities could help predict the regioselectivity of such reactions. However, hydricity and BDEs both follow the tertiary *>* secondary *>* primary reactivity pattern; it is thus not clear whether hydricities offer an advantage over BDEs. Furthermore, for reactions involving bulky catalysts and/or functional groups the regioselectivity will also be dictated by the steric accessibility of the C–H site (Figure 1).

In this paper we present a quantum chemistry (QM)-based workflow for the automatic prediction of hydricities. We use this QM workflow to create a training set for an ML-based hydricity predictor and give a few examples of how the method can be used to rationalise the regioselectivity of a diverse set of electron-rich C–H functionalisation reactions.

## Methods

### Datasets

To validate our QM workflow, we use a dataset of 35 experimental C–H hydricities in dimethyl sulfoxide (DMSO, 26 compounds) and acetonitrile (MeCN, 9 compounds) from Parker and co-workers [7]. As 35 C–H hydricities are insufficient to train an ML model, we use the same QM dataset from our previous paper [3] and combine it with the experimental dataset to calculate QM C–H hydricities. After removing compounds that failed the QM workflow (see next section), the total QM dataset consists of 740 compounds and 3278 hydricities and is used to train ML models.

### Quantum chemistry-based workflow

Following our previous work [3,8–11], we present a fully automated QM-based workflow that computes C–H hydricities. DMSO was chosen as solvent as most compounds (26/35) were experimentally measured in that medium. Hydride abstraction is carried out at each unique site by converting a SMILES string into a RDKit molecule object, producing a list of SMILES for each C–H bond. We generate min(1 + 3*n*_rot_, 20) conformers for each SMILES using RDKit (v.2023.09.3) [12], where (*n*_rot_) represents the number of rotatable bonds. Each conformer undergoes optimization in dimethyl sulfoxide (DMSO, ε = 47.2) using the GFN-FF-xTB [13] force field and the analytical linearised Poisson–Boltzmann (ALPB) [14] as the implicit solvation model. We then remove conformers with relative energies above 3 kcal/mol and select unique conformers by taking the centroids of a Butina clustering using pairwise heavy-atom root mean square deviation (RMSD) with a threshold of 0.5 Å [12,15].

To identify the lowest-energy conformer, we subsequently re-optimise the remaining conformers in DMSO with the semiemprical quantum chemistry method GFN2-xTB[16] and apply the ALPB implicit solvation model. We then conduct re-optimization in ORCA (v.5.0.4) [17–18], using the composite electronic structure method r^2^SCAN-3c [19] and the conductor-like polarizable continuum model (CPCM) [20] as the implicit solvation model. r^2^SCAN-3c is chosen as the optimal functional based on a benchmark study that evaluates the accuracy of different levels of theory, ranging from semiempirical methods (xTB [16]), composite electronic structure methods (r^2^SCAN-3c [19]) to DFT methods (M06-2X[21], CAM-B3LYP [22–23]). All methods are evaluated either as single-point calculations or optimization and frequency calculations. Refer to section 1 in Supporting Information File 1 for more details. Hereafter, we check the geometries for imaginary frequencies and use the total thermal energy at 298.15 K. Following a similar approach from our previous paper for C–H p*K*_a_ values [3], we compute the hydricity through the direct hydride transfer reaction, 
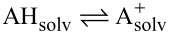
; see Equation 1.


[1] $$\Delta G^{{}^{\circ}}=G^{{}^{\circ}}\left({\text{A}}_{\text{solv}}^{+}\right)-G^{{}^{\circ}}\left({\text{AH}}_{\text{solv}}\right).$$

For each set of C–H sites in a molecule, we determine the minimum hydricity (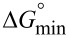). Hereafter, we assume a linear relationship between the experimental hydricity and 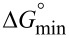 as this assumption allows us to derive the empirical constants *a* and *b* and correct any systematic errors, such as the hydride (H^−^) ion; see Equation 2, where Δ*G*° is replaced by 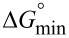. After retrieving the empirical constants *a* and *b*, we can determine the QM-computed hydricities for all C–H sites using Equation 2:


[2] $$\text{hydricity}=a\cdot \Delta G^{{}^{\circ}}+b.$$

Because *G*°(H^−^)_solv_ is constant across substrates, it is absorbed into the fitted intercept *b*.

## Machine Learning

### The feature descriptor

Recent research shows that the atomic descriptors introduced by Finkelmann et al. [24–25], using charge model 5 (CM5) atomic charges [26], is an excellent representation of atoms in molecules as the feature descriptor for ML models to predict various properties. These properties encompass the site of metabolism [25,27], the strengths of hydrogen bond donors and acceptors [28–30], the regioselectivity of electrophilic aromatic substitution reactions [10], C–H p*K*_a_ values [3], and electro- and nucleophilicity [31]. Building on the methodology from Finkelmann et al. [24–25], Ree et al. [10] and our previous work [3], we utilise the automated approach to compute CM5 atomic charges from semiempirical tight-binding (GFN1-xTB [32]) calculations. We modify the workflow to enhance the accuracy of the computed CM5 atomic charges. Instead of generating a single random conformer, we produce 20 random conformers from a SMILES string and optimise the structure with molecular mechanics force fields (MMFF) [33] using RDKit [12]. The CM5 atomic charges of the lowest-energy conformer are then used to generate atomic descriptors based on sorting the CM5 charges for a given atom of the input SMILES string. We have updated the sorting algorithm to improve adherence to the Cahn–Ingold–Prelog rules. Furthermore, we use a shell radius of 3 to describe the local environment better and speed up the training of our ML model, but at the cost of a slight decrease in accuracy. Refer to section 4 in Supporting Information File 1 for more details. The percentages of buried volumes of the C atoms are computed using MORFEUS [34].

### Data preparation and hyperparameter optimization

Following the methodology by Ree et al. and our previous work [3,10], we use the Optuna framework (v.3.3.0) [35] for hyperparameter optimization of two ML models, that is, a LightGBM regressor and classifier [36]. We employ the tree-structured Parzen estimator Bayesian method to avoid discouraging trails.

For regression, the target values are QM-computed hydricities. For binary classification, which aims to predict the site with the lowest QM-computed hydricity, we assign labels as “1” for the site with the lowest hydricity and “0” for all others. A cutoff is introduced, where hydricities within +1 or +2 kcal/mol of the lowest value are also labeled “1”. Refer to section 5 in Supporting Information File 1 for more details. Due to class imbalance (with “0”s far outnumbering “1”s), we use the *scale_pos_weight* hyperparameter. To illustrate the imbalance, we train a zero-rate classifier (null model) that predicts all sites as “0” and a random classifier to establish the theoretical baseline of model performance.

We split the dataset (740 compounds; 3278 hydricities) by compound into a training set (80%; 595 compounds; 2607 hydricities) and a test set (20%; 145 compounds; 671 hydricities). For each ML model, we perform fivefold random shuffled cross-validation. We then train the final ML model on the entire training set and evaluate it on the test set, selecting the best-performing model.

## Results and Discussion

### Computing C–H hydricities

**Figure 2:**
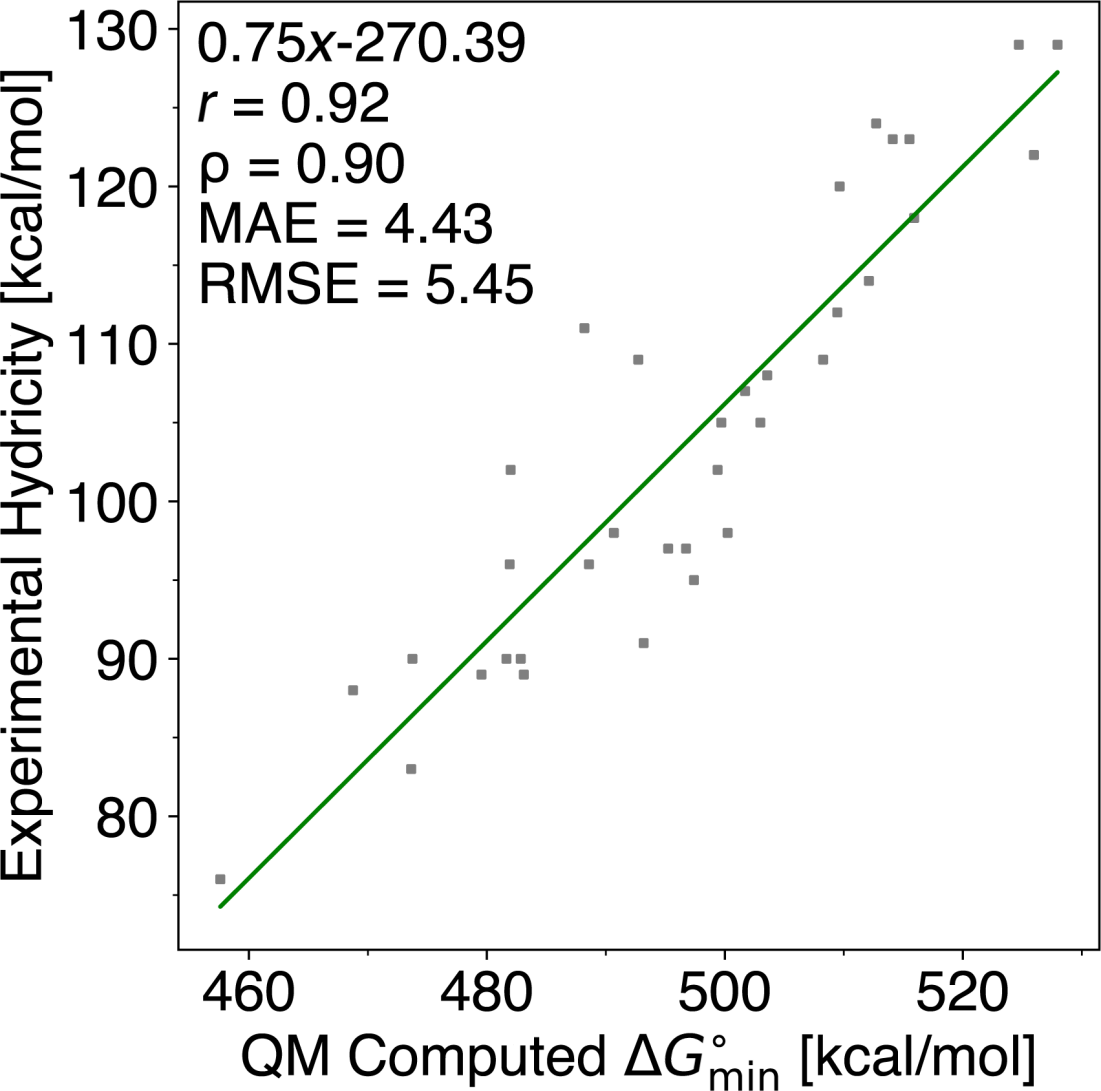
Correlating QM-computed 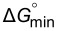 values and experimental hydricities for 35 compounds [7]. *r*: Pearson correlation coefficient; ρ: Spearman’s rank correlation coefficient; MAE: mean absolute error; RMSE: root mean squared error. QM calculations are carried out at the r^2^SCAN-3c level of theory.

In section “Quantum chemistry-based workflow”, we determine the empirical values *a* and *b* in Equation 2. For each set of C–H sites in a molecule, we extract the computed value of 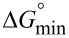 and fit it against experimental hydricities, obtaining a mean absolute error (MAE) of 4.43 kcal/mol and a root mean squared error (RMSE) of 5.45 kcal/mol (see Figure 2). The relatively large discrepancy between prediction and experiment could, in part, derive from experimental error.

These values ultimately trace back to the electrochemical thermochemical-cycle approach of Cheng, Handoo, and Parker [7], where solution hydride affinities are obtained from a cycle combining acidity data and redox potentials. Importantly, Cheng et al. emphasise that standard (reversible) electrode potentials are required for thermochemical cycles, but that experimentally measured potentials are often not reversible; thus, corrections may be needed to account for kinetic contributions. For relatively stable carbocations, the relevant potentials can be measured directly from carbocation salts (cyclic voltammetry), which is expected to give lower uncertainty. For less stable primary/secondary systems, precisely those that broaden the dataset beyond “classical” persistent carbocations, Cheng et al. instead determined potentials for photochemically generated radicals using photomodulation AC voltammetry (PACV). In this regime, Cheng et al. explicitly report an uncertainty of ±50 mV for the PACV-derived potentials. This alone corresponds to an uncertainty of the order of ≈1 kcal/mol per 50 mV term in the free-energy expression (and can compound when multiple electrochemical terms contribute); this means that an uncertainty of several kilocalories per mole is plausible for the less stable/transient subset. Consistent with this, Parker/Cheng highlight a higher uncertainty for the toluene/fluorene/9-methylanthracene-derived cations, and we observe that most of the large deviations occur in benzyl/anthracene-like cases as discussed in section 2 in Supporting Information File 1. We thus expect the DFT predictions to be significantly more accurate than the reported error, especially for chemically similar groups. Note that the linear scaling parameters derived from these experimental values do not change the ranking of hydricities within a molecule, which is the primary use case for our method as described below.

**Figure 3:**
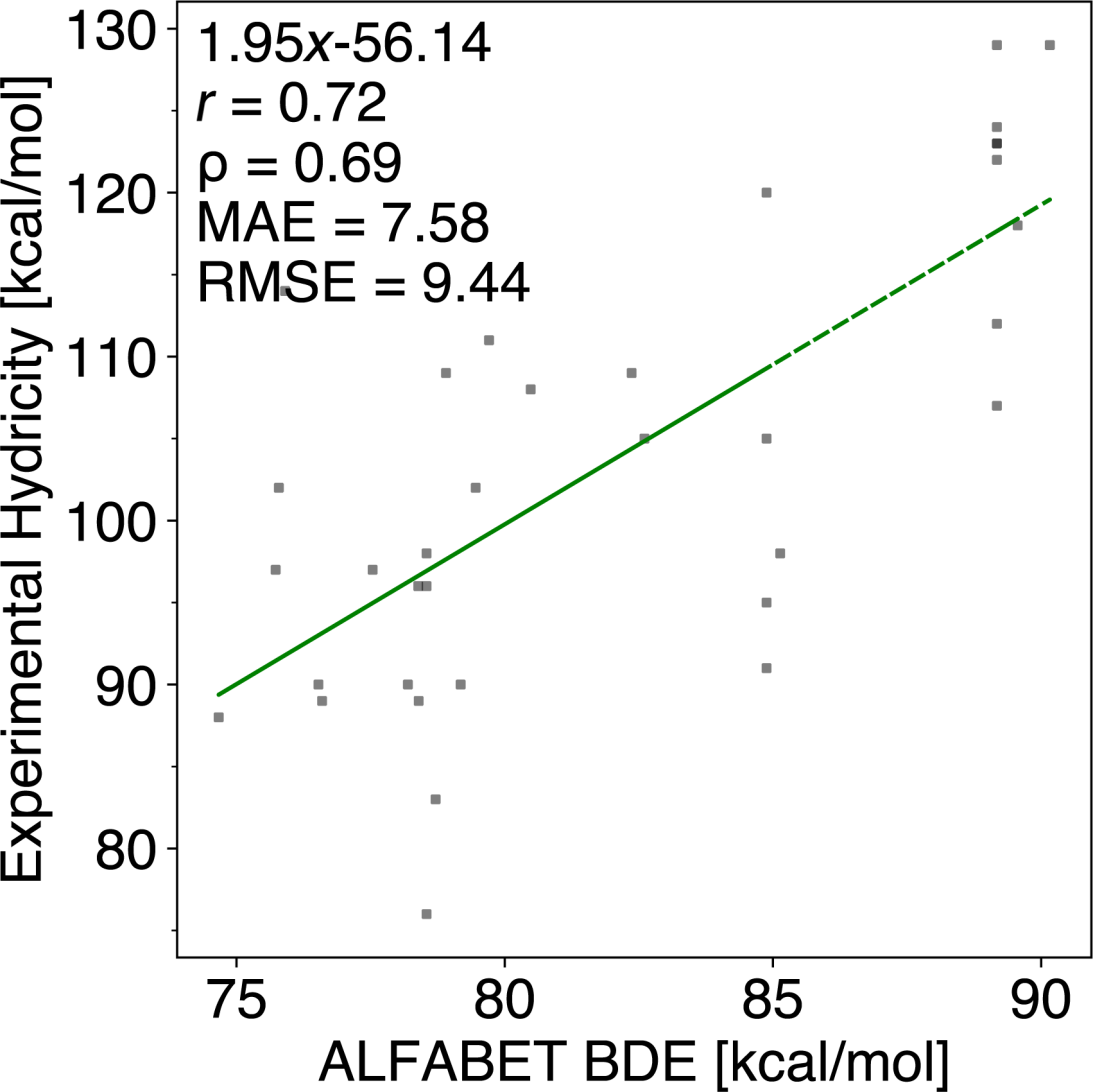
Correlating ML-predicted BDE values using ALFABET and experimental hydricities for 35 compounds [7]. *r*: Pearson correlation coefficient; ρ: Spearman’s rank correlation coefficient; MAE: mean absolute error; RMSE: root mean squared error.

We convert all computed Δ*G*° values into QM-computed hydricities (740 compounds; 3278 hydricities) using the derived linear regression. Outliers with differences above 6 kcal/mol are primarily anthracene derivatives and benzyl C–H bonds, suggesting potential systematic errors in QM computations or experimental measurements. Radical and cation stabilities generally follow the trend: tertiary *>* secondary *>* primary methyl carbon stability. Our study also explores the correlation between hydricities and BDEs. Using the ALFABET model developed by Paton and co-workers to predict BDEs [4–5,37], we find that our QM-computed hydricities exhibit a better correlation with experimental data compared to BDEs, with an MAE of 7.58 kcal/mol and an RMSE of 9.44 kcal/mol, see Figure 3. Refer to section 3 in Supporting Information File 1 for more details.

### Machine learning models for predicting C–H hydricity

**Figure 4:**
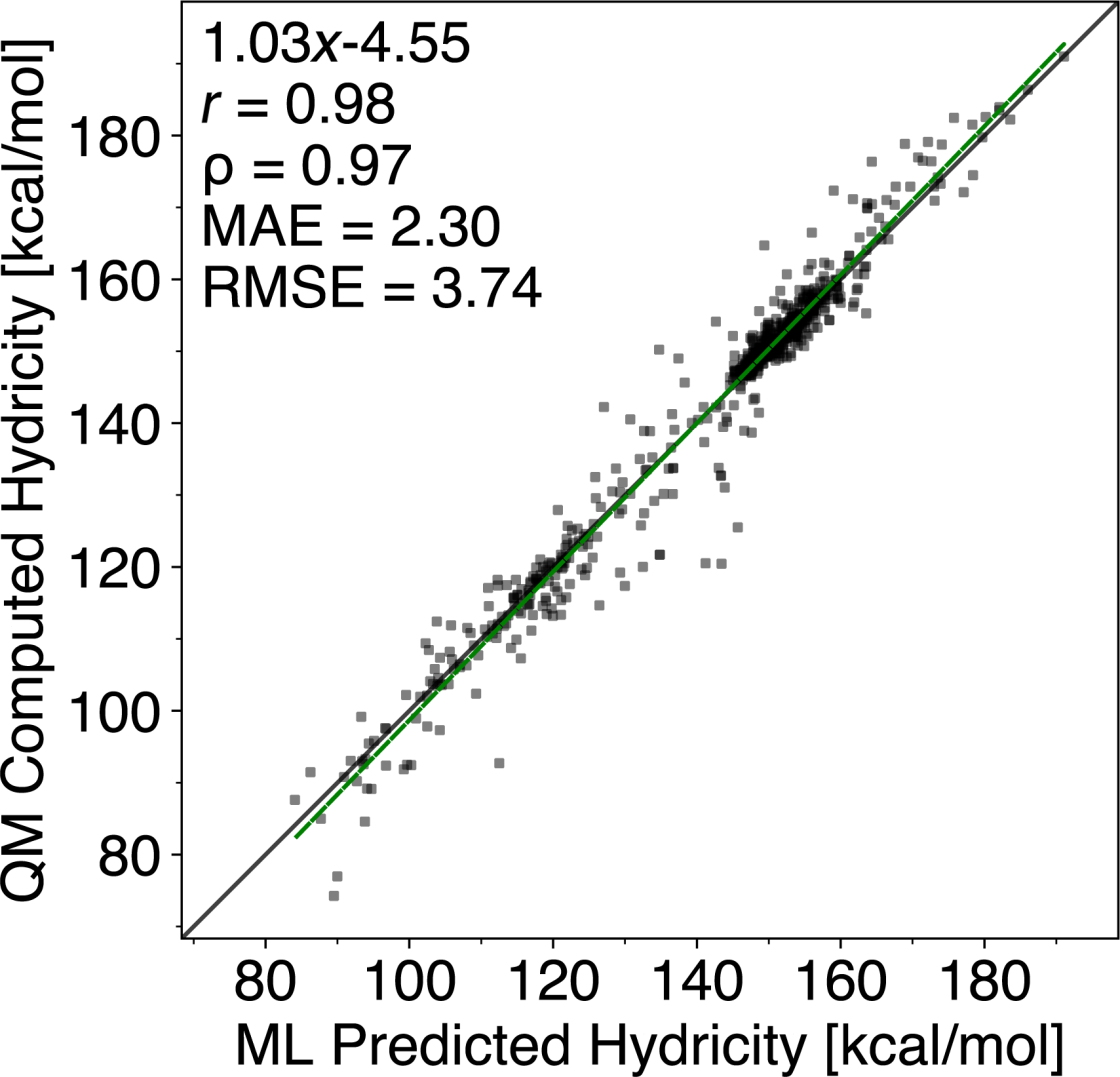
ML-predicted hydricities vs QM-computed hydricities of the held-out test set (145 compounds; 671 hydricities). *r*: Pearson correlation coefficient; ρ: Spearman’s rank correlation coefficient; MAE: mean absolute error; RMSE: root mean squared error. All predictions are carried out using the best ligthGBM regressor. All calculations are carried out at the r^2^SCAN-3c level of theory.

To learn and predict C–H hydricities, we train a LightGBM regressor with our QM dataset containing QM-computed hydricities (740 compounds; 3278 hydricities). Then, we correlate and compare the ML-predicted hydricities and the QM-computed hydricities and achieve an MAE and an RMSE of 2.30 and 3.74 kcal/mol, respectively, for the held-out test set (145 compounds; 671 hydricities), as illustrated in Figure 4.

For the ML-predicted hydricities that are not correlating well with the QM-computed hydricities, we find C–H sites where the positive charge is stabilised by resonance. We speculate that the ML model has seen few examples of the C–H sites that are outliers, and a more extensive training set would be advantageous. ALFABET is also compared against the test set, achieving an MAE and an RMSE of 6.64 and 9.49 kcal/mol, respectively. Refer to section 5.1 in Supporting Information File 1 for more details.

### Hydricity, BDE, and regioselectivity

**Figure 5:**
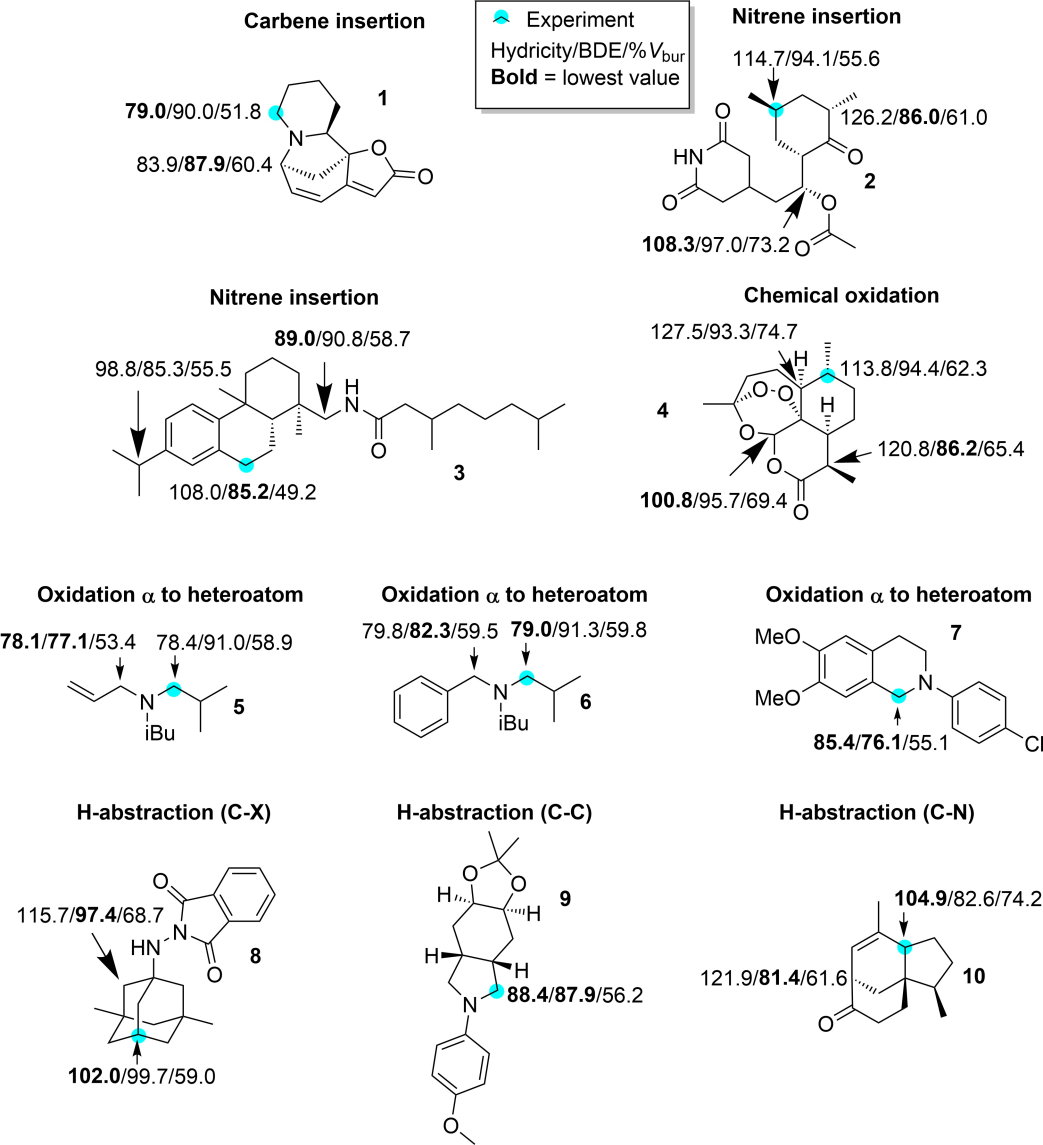
Examples of functionalisation of electron-rich C–H bonds via innate insertion or H-abstraction taken from Cernak and co-workers [6]. The experimentally observed functionalisation sites are marked with dots. The ML-predicted hydricity/bond dissociation energy/percentage of buried volume of the C atom are given for select atoms (the first two in units of kcal/mol). The numbers in bold represent the lowest value of hydricity and BDE computed for the molecule.

In order to test whether ML-predicted hydricities can be used to rationalise the regioselectivity of electron-rich C–H functionalisation reactions, we test nine reactions highlighted by Cernak et al. [6] and add an additional reaction (compound **6**, see below). For each reactant, we compute the hydricities as well as the BDEs using the ML method developed by Paton and co-workers [4–5] and the percentage of buried volume of the relevant C atoms (%*V*_bur_). Many of these transformations are not formal hydride transfers; however, they commonly involve buildup of positive charge (e.g., via metal–carbene/nitrene insertion transition states, radical cations, or polar hydrogen atom transfer). Hydricity could therefore serve here as an empirical reactivity descriptor rather than a mechanistic assignment. The results are shown in Figure 5.

#### Compound **1**

He et al. [38] reported a dirhodium(II)-catalysed intermolecular C–H insertion into securinenine (compound **1**) to generate a C–C bond. The reaction occurs at the site with the lowest predicted hydricity, but only the third-lowest BDE.

#### Compounds **2** and **3**

Roizen et al. [39] reported dirhodium(II)-catalysed nitrene insertion into cycloheximide (compound **2**). The reactive site does not have the lowest hydricity nor BDE. Instead, the correct site has the second lowest hydricity. The site with lowest hydricity is sterically very crowded with %*V*_bur_ = 73%. While a reaction occurs at an even more crowded site in compound **10** (%*V*_bur_ = 74.5%), this reaction is catalysed by a significantly less bulky catalyst (a Cu(II) salt) (see below). In contrast to the hydricity, the reactive site has only the fifth-smallest BDE.

However, nitrene insertion into compound **3** [40] is better predicted by BDE, although the two lowest BDEs are within 0.1 kcal/mol of each other. As noted by Cernak et al. [6], reaction selectivity of this reaction “has a strong dependence on the structure of the nitrene precursor, highlighting the caution that must be used when applying simple measures of selectivity prediction in a complex setting.”

#### Compound **4**

Vermeulen et al. [41] reported the Fe(DPD)-catalysed oxidation of (+)-artemisinin (compound **4**). Similarly to nitrene insertion into cycloheximide (compound **2**), the reactive site does not correspond to the site with lowest predicted hydricity nor BDE. Rather, the reacting site has the second lowest hydricity and the third-lowest BDE. However, the site with lowest hydricity has a relatively large %*V*_bur_ (69.4%) and may be sterically inaccessible, while the site with the second-lowest hydricity is less sterically hindered (%*V*_bur_ = 62.3%). For comparison, the site with the lowest BDE has an intermediate value for %*V*_bur_ of 65.4%.

#### Compounds **5** and **6**

Allen and Lambert [42] reported the tropylium ion-mediated α-cyanation of amines, including compounds **5** and **6**. In the case of compound **5**, the observed site of reaction is the site with the second lowest hydricity and BDE. However, the hydricity of the reactive site is within only 0.3 kcal/mol of the site with the lowest hydricity, while the corresponding BDE is significantly higher. Thus, based on the BDE one would confidently predict the incorrect reactive site, while for the hydricity two possible reaction sites would be identified, one of which is correct. For the closely related compound **6**, hydricity more clearly identifies the most reactive site, in contrast to the BDE.

#### Compound **7**

Bergonzini et al. [43] reported the Ru-catalysed photoredox activation, followed by anion-binding-catalysed functionalisation to form β-amino esters. Here, the reacting atom is the one with the lowest hydricity and BDE.

#### Compounds **8–10**

Schmidt et al. [44] reported the visible light-catalysed bromination of compound **8**, Kamon et al. [45] reported the light-catalysed C–H carbamoylation of the *cis*-fused azabicyclo[4.3.0]nonane derivative **9**, and Overman and Tomasi [46] reported the Cu(II) salt-catalysed addition of a *N*-tosyl group to the C–C double bond in compound **10** in conjunction with an H-abstraction, to form a new C–C double bond. In all three cases, the reaction occurs at the site with lowest hydricity and not lowest BDE, with the exception of compound **9**, where they coincide.

In summary, the site with lowest hydricity corresponds to the correct reactive site in six of the ten molecules considered here. In two additional compounds, the site with the lowest hydricity is likely sterically inaccessible, and the reaction occurs at the site with the second lowest hydricity. So, in eight out of ten cases the regioselectivity can be correctly predicted by using hydricity and steric accessibility. In one of the two remaining cases the hydricity predicts two reactive sites, where one of them is the correct site. So hydricity only fails to correctly predict the reactive site in one case (compound **3**).

In contrast, the BDE can correctly predict the reactive site in two cases (that are also predicted correctly using hydricities); in a third case, BDE predicts two reactive sites, where one of them is the correct site. Not surprisingly, the two reactions where the use of BDEs lead to the correct predictions are photocatalysed and generally thought to involve radical formation.

In general, the sites of lowest hydricity and BDE only coincide in three cases (compounds **5**, **7**, and **9**) indicating that hydricities offer a valuable measure of reactivity in addition to BDEs for these types of reactions.

## Conclusion

We have developed HAlator, an automated QM-based workflow for computing C–H hydricities, benchmarked against 35 experimental values in DMSO to yield an MAE of 4.43 kcal/mol and an RMSE of 5.45 kcal/mol. Using the derived linear correlation, we generated a dataset of 3,278 C–H sites from 740 molecules to train a LightGBM regressor based on CM5 atomic charge descriptors. The resulting ML model reproduces QM-computed hydricities with an MAE of 2.30 kcal/mol and an RMSE of 3.74 kcal/mol on a held-out test set of 671 C–H sites.

When tested on ten representative literature examples of electron-rich C–H functionalisation, hydricity combined with steric accessibility correctly identified the experimental reactive site in eight out of ten cases, compared to three out of ten for BDEs alone. Notably, hydricity provided unambiguous predictions in six out of ten cases, and in two further cases the correct site was the second-lowest hydricity but the most sterically accessible. BDEs only outperformed hydricity in one case (compound **3**).

These results quantitatively demonstrate that hydricity is a valuable and complementary descriptor to BDEs for regioselectivity prediction in electron-rich C–H functionalisation. Future work will focus on expanding the training dataset beyond 3,278 C–H sites, integrating explicit steric and electronic descriptors, and improving predictive accuracy for sterically demanding carbene insertion reactions, where all current methods underperform.

While the ten literature examples discussed here provide promising proof-of-concept evidence, establishing the general utility of hydricity for regioselectivity prediction will require validation against a much larger and more chemically diverse dataset. In particular, steric accessibility is inherently catalyst-dependent, and its predictive integration will need to account for variations in catalyst size, shape, and approach geometry. Large-scale benchmarking across multiple catalyst classes will therefore be essential to determine the robustness and transferability of hydricity-guided predictions.

The model is made available at regioselect.org together with a host of other reactivity predictors.

## Supporting Information

File 1 Additional computational data.

## Data Availability

The code for the automated workflow and results of the analyzed data are available at https://github.com/jensengroup/HAlator. Additional data is available at https://sid.erda.dk/sharelink/coKwQQzlzr.
