# Supplementary material for: Computational prediction of C–H hydricities and their use in predicting the regioselectivity of electron-rich C–H functionalisation reactions
Source: Beilstein J Org Chem. 2026 Apr 17;22:603–10. doi: 10.3762/bjoc.22.46 (PMC13159236; doi:10.3762/bjoc.22.46)
Supplement: File 1 — Additional computational data. [file Beilstein_J_Org_Chem-22-603-s001.pdf]

BEILSTEIN JOURNAL OF ORGANIC CHEMISTRY

Supporting Information

for

Computational prediction of C–H hydricities and their use in predicting the regioselectivity of electron-rich C–H functionalisation reactions

Rasmus M. Borup, Nicolai Ree and Jan H. Jensen

Beilstein J. Org. Chem. **2026,** *22,* 603–610. doi:10.3762/bjoc.22.46

Additional computational data

License and Terms: This is a supporting information file under the terms of the Creative Commons Attribution License (https://creativecommons.org/licenses/by/4.0). Please note that the reuse, redistribution and reproduction in particular requires that the author(s) and source are credited and that individual graphics may be subject to special legal provisions.
The license is subject to the *Beilstein Journal of Organic Chemistry* terms and conditions: (https://www.beilstein-journals.org/bjoc/terms)

1. Benchmark study - computational methods

This benchmark study evaluates the computational effort and accuracy of single-point calculations or re-optimizations based on GFN2-xTB. These calculations utilize ORCA (v. 5.0.4)[1,2] with DMSO serving as the solvent. The following functionals and basis sets are used: composite electronic structure method r^{2}SCAN-3c[3], combined with its custom def2-mTZVPP/J basis set; the hybrid meta-GGA functional M06-2X[4] combined with the Karlsruhe triple-ζ basis set (def2-TZVP)[5,6]; the range-separated hybrid functional and dispersion D4 corrected CAM-B3LYP[7,8] combined with the Karlsruhe triple-ζ basis set (def2-TZVPPD)[5,6]. The Conductor-like Polarizable Continuum Model (CPCM)[9] implicit solvation model is employed for all computations. Reoptimization with r^{2}SCAN-3c from GFN2-xTB shows overall best performance as demonstrated in S1, S1, and S2. Figures S2–S8 shows linear correlations between ΔG°_{min} against experimental hydricities for the different QM methods, for the dataset from Parker and coworkers[10] (35 compounds).

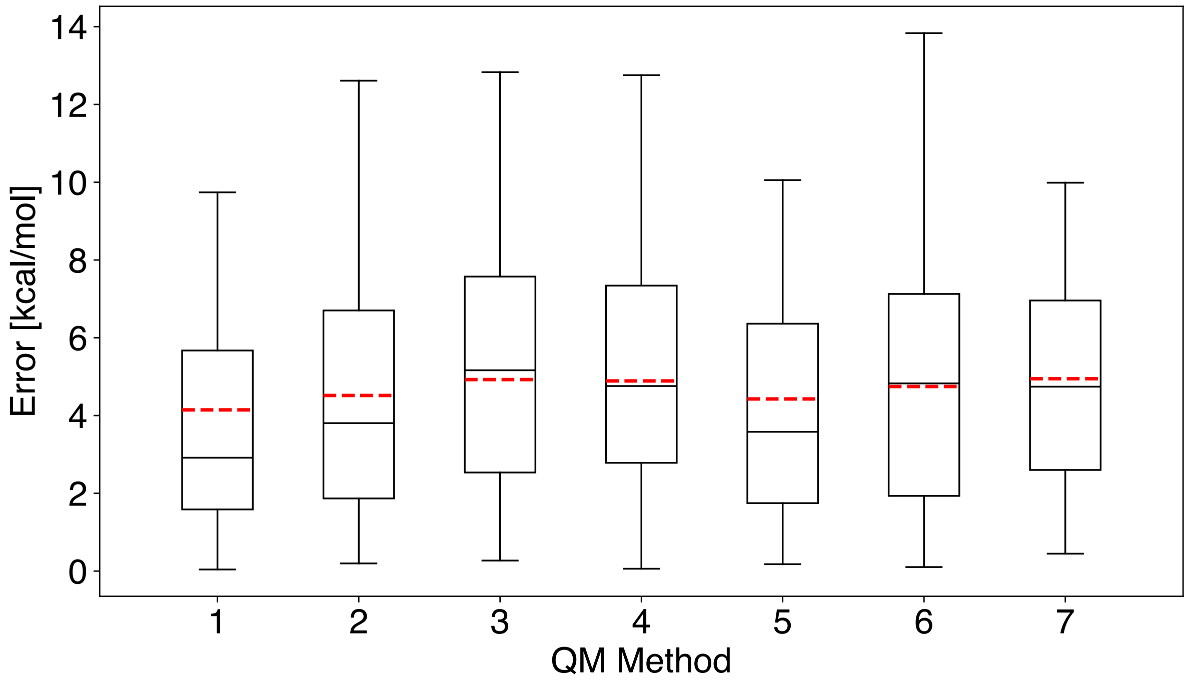

Figure S1: Absolute errors (kcal/mol) for different levels of theory using the data from Parker and coworkers[10] (35 compounds). (1) GFN2-xTB ALPB(DMSO); (2) r^{2}SCAN-3c SMD(DMSO)/GFN2-xTB ALPB(DMSO); (3) M06-2X/def2-TZVP CPCM(DMSO)/GFN2-xTB ALPB(DMSO); (4) CAM-B3LYP D4/def2-TZVPPD CPCM(DMSO)/GFN2-xTB ALPB(DMSO); (5) r^{2}SCAN-3c CPCM(DMSO))//GFN2-xTB ALPB(DMSO); (6) M06-2X/def2-TZVP CPCM(DMSO)//GFN2-xTB ALPB(DMSO); (7) CAM-B3LYP/def2-TZVPPD CPCM(DMSO)//GFN2-xTB ALPB(DMSO). The red line indicates the mean absolute error.

Table S1: Error metrics (kcal/mol) for different levels of theory using 35 compounds[10]. (1) GFN2-xTB ALPB(DMSO); (2) r^2SCAN-3c SMD(DMSO)/GFN2-xTB ALPB(DMSO); (3) M06-2X/def2-TZVP CPCM(DMSO)/GFN2-xTB ALPB(DMSO); (4) CAM-B3LYP D4/def2-TZVPPD CPCM(DMSO)/GFN2-xTB ALPB(DMSO); (5) r^2SCAN-3c CPCM(DMSO))//GFN2-xTB ALPB(DMSO); (6) M06-2X/def2-TZVP CPCM(DMSO)//GFN2-xTB ALPB(DMSO); (7) CAM-B3LYP/def2-TZVPPD CPCM(DMSO)//GFN2-xTB ALPB(DMSO).

	1	2	3	4	5	6	7
		Single Point			Reoptimization		
	GFN2-xTB	r^2SCAN-3c	M06-2X	CAM-B3LYP D4	**r^2SCAN-3c**	M06-2X	CAM-B3LYP D4
mean	4.14	4.52	4.92	4.89	4.43	4.75	4.95
std	3.69	3.11	3.10	3.18	3.23	3.13	3.13
min	0.04	0.20	0.27	0.06	0.18	0.10	0.45
25%	1.59	1.87	2.53	2.78	1.75	1.93	2.60
50%	2.92	3.80	5.16	4.76	3.58	4.83	4.74
75%	5.67	6.70	7.57	7.34	6.36	7.13	6.96
max	17.57	12.61	12.83	12.75	13.70	13.83	13.83

Table S2: Summary of the benchmark study for the dataset from Parker and coworkers[10] (35 compounds). r^2: coefficient of determination; ρ: Spearman's rank correlation coefficient; MAE: mean absolute error; RMSE: root mean squared error; reg: linear regression.

Functional	Basis set	sp / opt-freq	MAE	RMSE	r	ρ	reg
GFN2-xTB	-	-	4.14	5.51	0.91	0.89	$0.62x - 251.18$
r^2SCAN-3c	def2-mTZVPP/J	single-point	4.52	5.46	0.92	0.91	$0.75x - 273.28$
M06-2X	def2-TZVP	single-point	4.92	5.80	0.91	0.90	$0.78x - 289.32$
CAM-B3LYP D4	def2-TZVPPD	single-point	4.89	5.81	0.90	0.89	$0.76x - 280.60$
**r^2SCAN-3c**	def2-mTZVPP/J	opt-freq	4.43	5.45	0.92	0.90	$0.75x - 270.39$
M06-2X	def2-TZVP	opt-freq	4.75	5.66	0.91	0.90	$0.78x - 288.19$
CAM-B3LYP D4	def2-TZVPPD	opt-freq	4.95	5.83	0.90	0.89	$0.76x - 275.11$

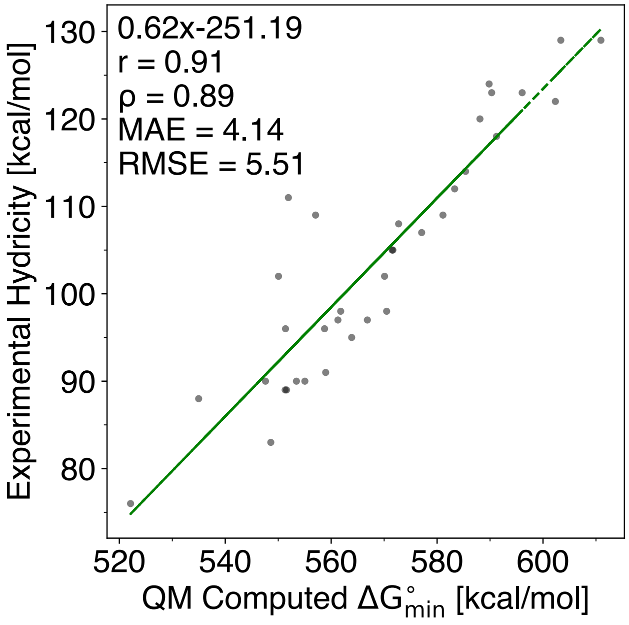

Figure S2: Correlating QM computed ΔG°_{min} values and experimental hydricities for 35 compounds[10]. r: Pearson correlation coefficient; ρ: Spearman's rank correlation coefficient; MAE: mean absolute error; RMSE: root mean squared error. QM calculations are carried out at the GFN2-xTB ALPB(DMSO) level of theory.

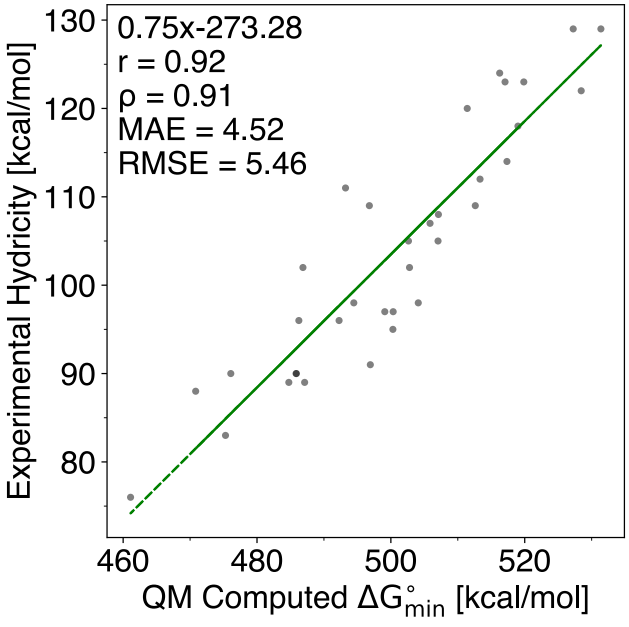

Figure S3: Correlating QM computed ΔG°_{min} values and experimental hydricities for 35 compounds[10]. r: Pearson correlation coefficient; ρ: Spearman's rank correlation coefficient; MAE: mean absolute error; RMSE: root mean squared error. QM calculations are carried out at the r^2SCAN-3c/def2-mTZVPP/J CPCM(DMSO)/GFN2-xTB ALPB(DMSO) level of theory.

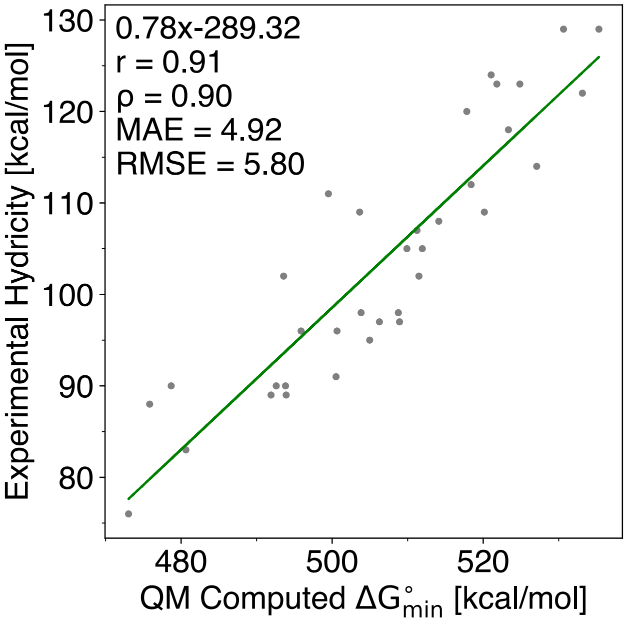

Figure S4: Correlating QM computed ΔG°_{min} values and experimental hydricities for 35 compounds[10]. r: Pearson correlation coefficient; ρ: Spearman's rank correlation coefficient; MAE: mean absolute error; RMSE: root mean squared error. QM calculations are carried out at the M06-2X/def2-TZVP CPCM(DMSO)/GFN2-xTB ALPB(DMSO) level of theory.

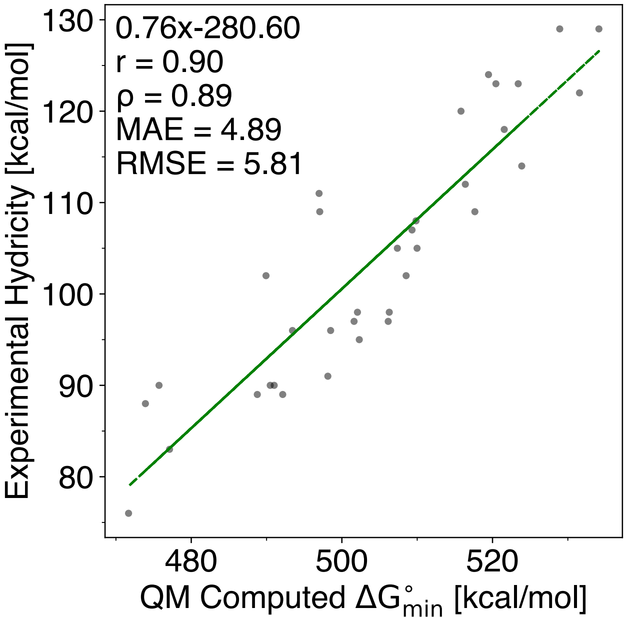

Figure S5: Correlating QM computed ΔG°_{min} values and experimental hydricities for 35 compounds[10]. r: Pearson correlation coefficient; ρ: Spearman's rank correlation coefficient; MAE: mean absolute error; RMSE: root mean squared error. QM calculations are carried out at the CAM-B3LYP/def2-TZVPPD CPCM(DMSO)/GFN2-xTB ALPB(DMSO) level of theory.

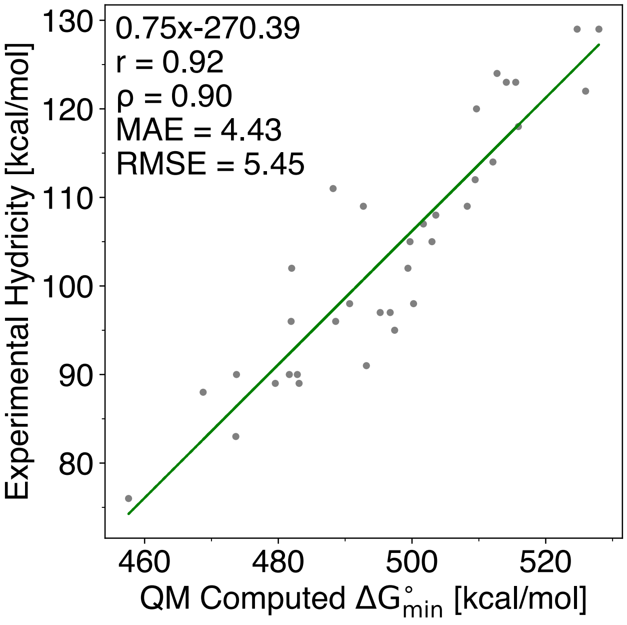

Figure S6: Correlating QM computed ΔG°_{min} values and experimental hydricities for 35 compounds[10]. r: Pearson correlation coefficient; ρ: Spearman's rank correlation coefficient; MAE: mean absolute error; RMSE: root mean squared error. QM calculations are carried out at the r^{2}SCAN-3c/def2-mTZVPP/J CPCM(DMSO)//GFN2-xTB ALPB(DMSO) level of theory.

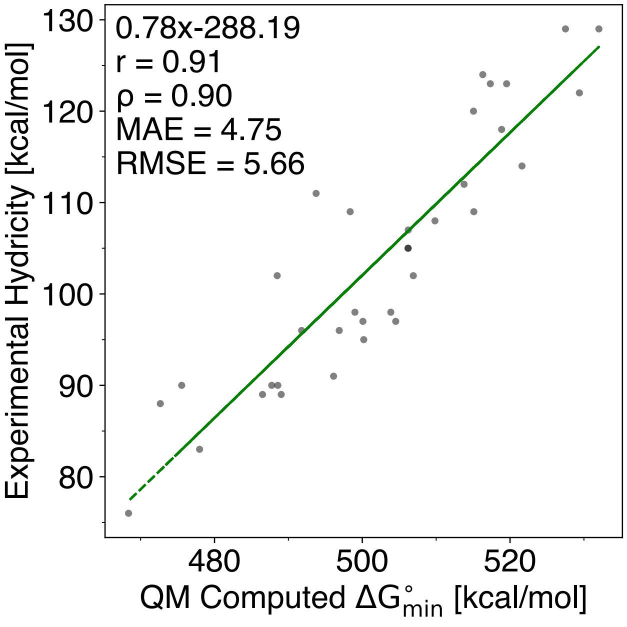

Figure S7: Correlating QM computed ΔG°_{min} values and experimental hydricities for 35 compounds[10]. r: Pearson correlation coefficient; ρ: Spearman's rank correlation coefficient; MAE: mean absolute error; RMSE: root mean squared error. QM calculations are carried out at the M06-2X/def2-TZVP CPCM(DMSO)//GFN2-xTB ALPB(DMSO) level of theory.

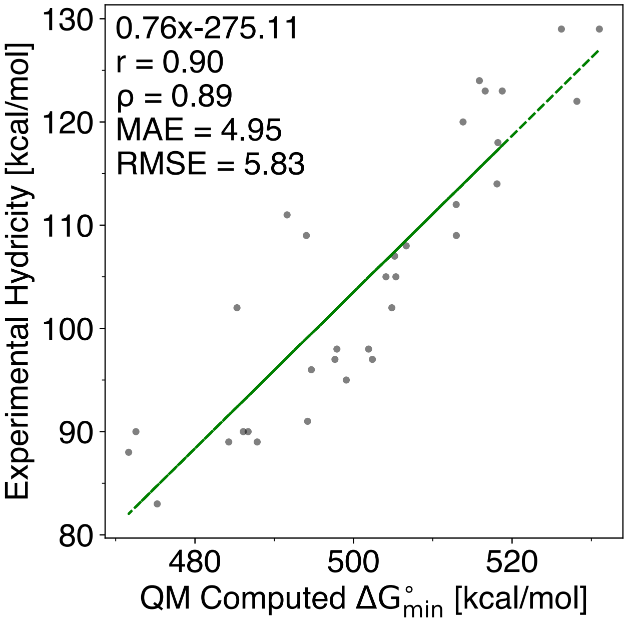

Figure S8: Correlating QM computed ΔG°_{min} values and experimental hydricities for 35 compounds[10]. r: Pearson correlation coefficient; ρ: Spearman's rank correlation coefficient; MAE: mean absolute error; RMSE: root mean squared error. QM calculations are carried out at the CAM-B3LYP/def2-TZVPPD CPCM(DMSO)//GFN2-xTB ALPB(DMSO) level of theory.

2. Outliers between experimental and QM hydricities

Of the 35 compounds, we find 10 compounds to have a difference in the hydricity above 6 kcal/mol between the experimental and QM computed values. We observe most of the outliers are anthracene derivates. From the experimental data, 7/8 of the anthracene derivatives have high errors. This may indicate either a systematic error from the QM part or in the experimental measurements. Notably, 9/10 outliers are at the benzyl C-H bond, see S9. Parker and coworkers also note that the experimental hydricities for toluenes, 9-substituted fluorenes, and α- or 10-substituted 9-methylanthracenes has a higher degree of uncertainty[10]. If the compounds are removed, We get a better correlation between the QM computed hydricities and the experimental hydricities. Still, we have chosen to keep them in our linear regression as the linear fit would be on a small number of compounds (25). See Figure S10 for the linear regression without outliers.

Ellis et al.[11] also find a discrepancy between experimentally measured hydricity for Bz-NADH from Parker and coworkers[12]. Here, Ellis et al. report a hydricity of 59±2 kcal/mol, whereas Parker and coworkers report a hydricity of 72 kcal/mol, a 13 kcal/mol difference in MeCN. Ellis et al. state that they get the same value (59±2 kcal/mol in MeCN) as Cheng et al.[13] (59.0 kcal/mol in DMSO). However, hydricities are known to be solvent dependent[14,15] and can change significantly from DMSO (DMSO, $\epsilon = 47.2$) to MeCN (MeCN, $\epsilon = 36.6$). Also, the articles use reference numbers from Parker and coworkers, making it difficult to justify what is correct. Based on those findings, we recognize that our experimental dataset may be prone to high inaccuracies. Still, we want to acknowledge the previous experimental

work from Parker and coworkers as it is the only first-hand (true) reference available. We encourage the experimental chemists to explore the hydricities of organic hydride donors further, broadening the knowledge in the field.

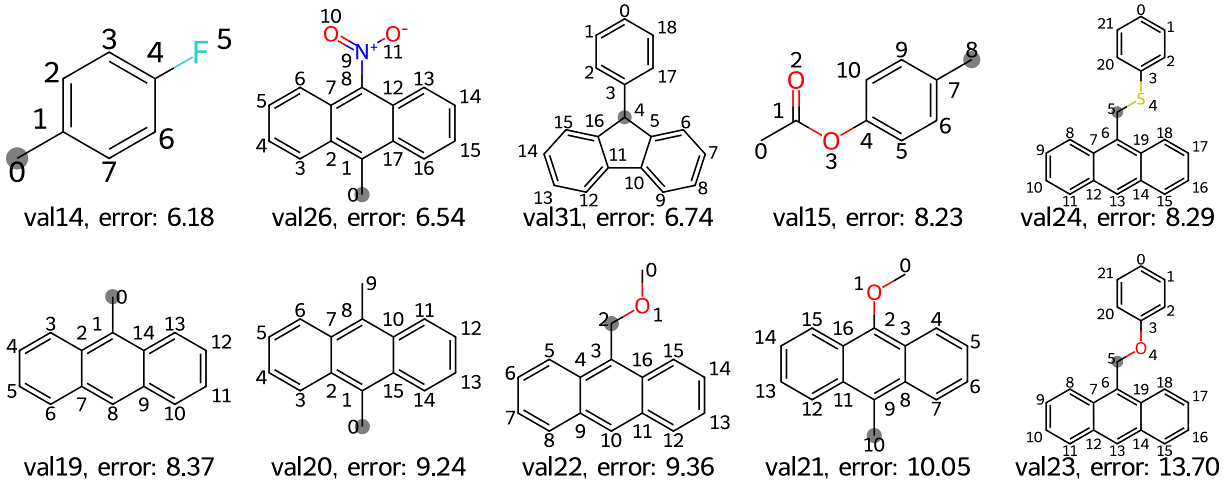

Figure S9: 10 outliers with an error of 6 kcal/mol or above between QM computed ΔG°_{min} values and experimental hydricities. Minimum QM computed site (GREY). QM calculations are carried out at the r^{2}SCAN-3c/def2-mTZVPP/J CPCM(DMSO)//GFN2-xTB ALPB(DMSO) level of theory.

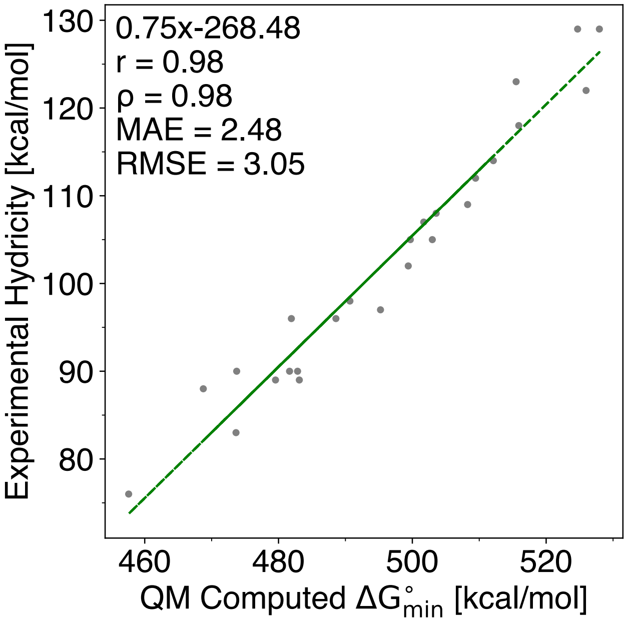

Figure S10: Correlating QM computed ΔG°_{min} values and experimental hydricities for 25 compounds, removing 10 outliers[10]. r: Pearson correlation coefficient; ρ: Spearman's rank correlation coefficient; MAE: mean absolute error; RMSE: root mean squared error. QM calculations are carried out at the r^{2}SCAN-3c/def2-mTZVPP/J CPCM(DMSO)//GFN2-xTB ALPB(DMSO) level of theory.

3. Correlating Hydricity with Bond Dissociation Energy

The same rule of thumb is applied for both the stability of radicals and cations, that the more substituted is the most stable tertiary>secondary>primary>>methyl. The thermodynamic stability of radicals is usually measured by the homolytic bond dissociation energy (BDE), whereas hydricity is the heterolytic bond dissociation energy to form a carbocation. Paton and coworkers have automated density functional theory (DFT) calculations at the M06-2X/def2-TZVP level of theory for 42,577 small organic molecules, resulting in 290,664 BDEs. Hereafter, A graph neural network (ALFABET) is trained on a subset of these results to achieve a mean absolute error of 0.58 kcal/mol (vs. DFT) for BDEs of unseen molecules. Lately, they also updated ALFABET, enabling a broader range of chemical space to be studied by this approach[16–18]. To see if there is a correlation between the experimental hydricities from Parker and coworkers[10] and the ALFABET BDE, we feed ALFABET with the compounds, leaving an MAE and RMSE of 7.58 and 9.44, respectively, S11. We also check if there is a relationship between our QM method for hydricities and ALFABET. We benchmark against the same functional (M06-2X) and basis set (def2-TZVP) as they use in the article but also benchmark against the best QM method (r^{2}SCAN-3c) from section 1. Benchmark study - computational methods are used in the supporting information. For S12, using the M06-2X functional, we find no correlation between QM computed hydricities and ALFABET BDEs (MAE of 4.56, RMSE of 5.05). However, for S13, using the functional r^{2}SCAN-3c, there is a good correlation with an MAE of 2.60 and RMSE of 3.38. We find it odd that the correlation is so bad for the functional M06-2X and, therefore, also try to

calculate BDE using our QM methods. Instead of abstracting a hydride, we now abstract a hydrogen, leaving us with a radical, $AH_{solv} \rightleftharpoons A^{\cdot}_{solv}$, see Equation 3. We leave the hydrogen ($H^{\cdot}$) out due to difficulties in validating its exact energy in solution.

$$\Delta G^{\circ} = G^{\circ}(A^{\cdot}_{solv}) - G^{\circ}(AH_{solv}) \tag{3}$$

From Figures S14 and S15, we find a good correlation between our QM methods and ALFABET. The best correlation is found when using the functional (M06-2X), where we obtain an MAE of 1.30 and RMSE of 1.79, see S14. Using our best QM method, we get an MAE of 1.56 and RMSE of 2.13, see S15. This result agrees as ALFABET is trained on DFT calculations using M06-2X as the functional. Lastly, we correlate our QM methods for QM computed hydricities and QM computed BDEs. Here, there is no correlation with an MAE of 6.56 and RMSE of 7.68 (M06-2X) and an MAE of 5.35 and RMSE of 5.96 (r^2SCAN-3c). We, therefore, conclude that our approach is valid by computing hydricities instead of BDEs as it gives the best correlation overall.

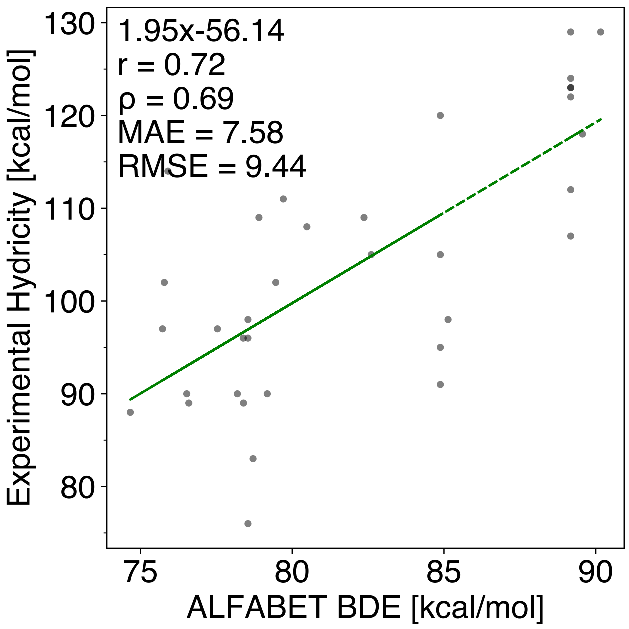

Figure S11: Correlating ML predicted BDE values using ALFABET and experimental hydricities for 35 compounds[10]. r: Pearson correlation coefficient; ρ: Spearman's rank correlation coefficient; MAE: mean absolute error; RMSE: root mean squared error.

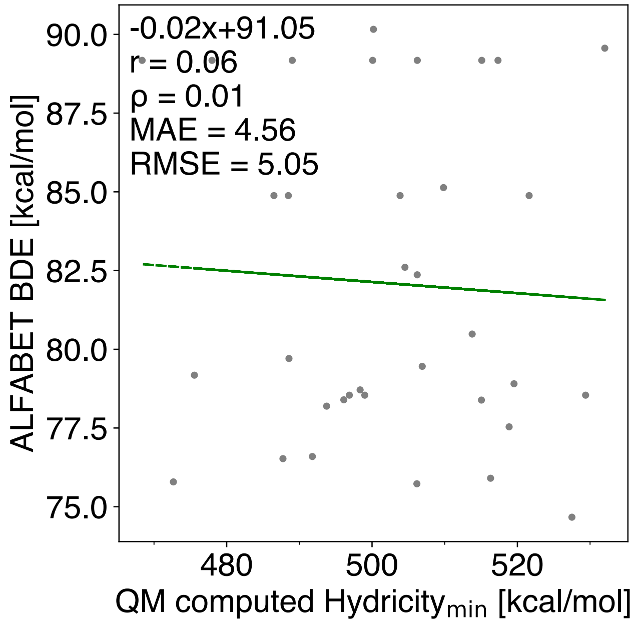

Figure S12: Correlating QM computed hydricity (ΔG°_{min}) values and ML predicted BDE values using ALFABET for 35 compounds[10]. r: Pearson correlation coefficient; ρ: Spearman's rank correlation coefficient; MAE: mean absolute error; RMSE: root mean squared error. QM calculations are carried out at the M06-2X/def2-TZVP CPCM(DMSO)//GFN2-xTB ALPB(DMSO) level of theory.

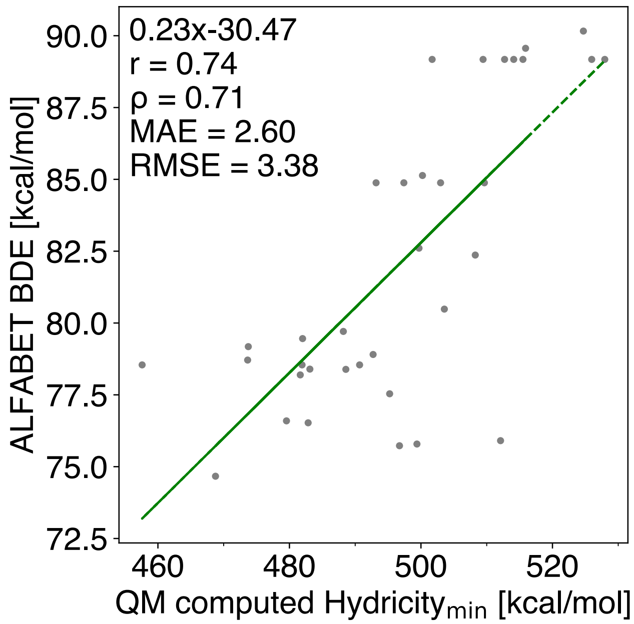

Figure S13: Correlating QM computed hydricity (ΔG°_{min}) values and ML predicted BDE values using ALFABET for 35 compounds[10]. r: Pearson correlation coefficient; ρ: Spearman's rank correlation coefficient; MAE: mean absolute error; RMSE: root mean squared error. QM calculations are carried out at the r^2SCAN-3c/def2-mTZVPP/J CPCM(DMSO)//GFN2-xTB ALPB(DMSO) level of theory.

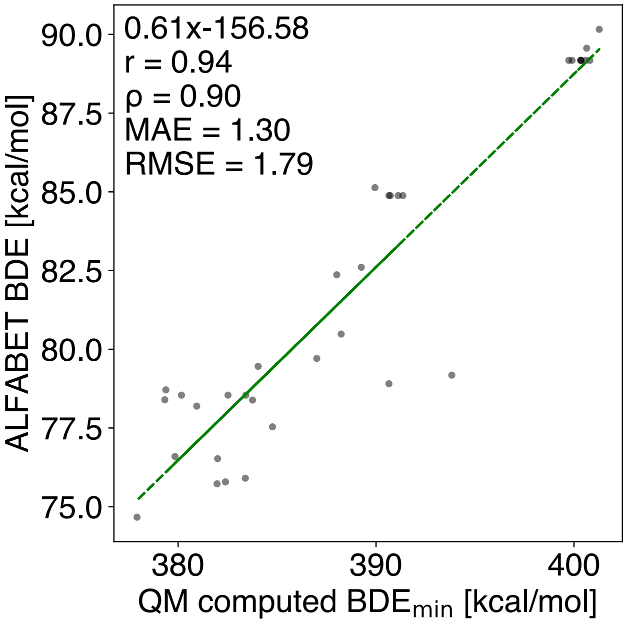

Figure S14: Correlating QM computed BDE (ΔG°_{min}) values and ML predicted BDE values using ALFABET for 35 compounds[10]. r: Pearson correlation coefficient; ρ: Spearman's rank correlation coefficient; MAE: mean absolute error; RMSE: root mean squared error. QM calculations are carried out at the M06-2X/def2-TZVP CPCM(DMSO)//GFN2-xTB ALPB(DMSO) level of theory.

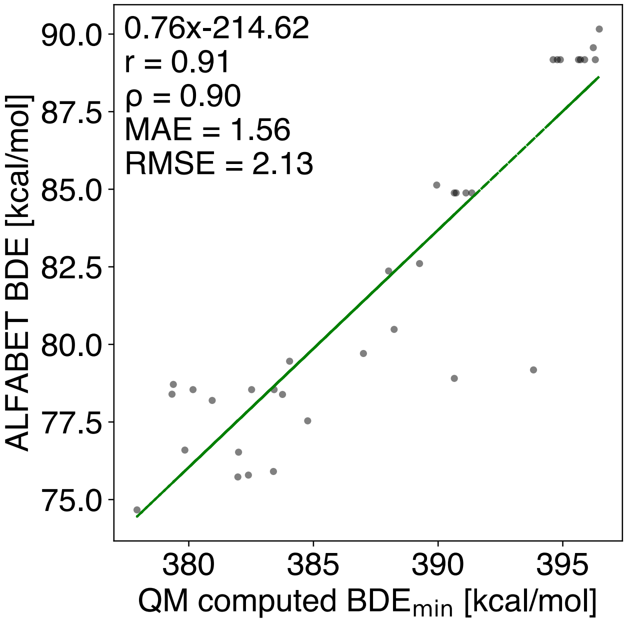

Figure S15: Correlating QM computed BDE (ΔG°_{min}) values and ML predicted BDE values using ALFABET for 35 compounds[10]. r: Pearson correlation coefficient; ρ: Spearman's rank correlation coefficient; MAE: mean absolute error; RMSE: root mean squared error. QM calculations are carried out at the r^{2}SCAN-3c/def2-mTZVPP/J CPCM(DMSO)//GFN2-xTB ALPB(DMSO) level of theory.

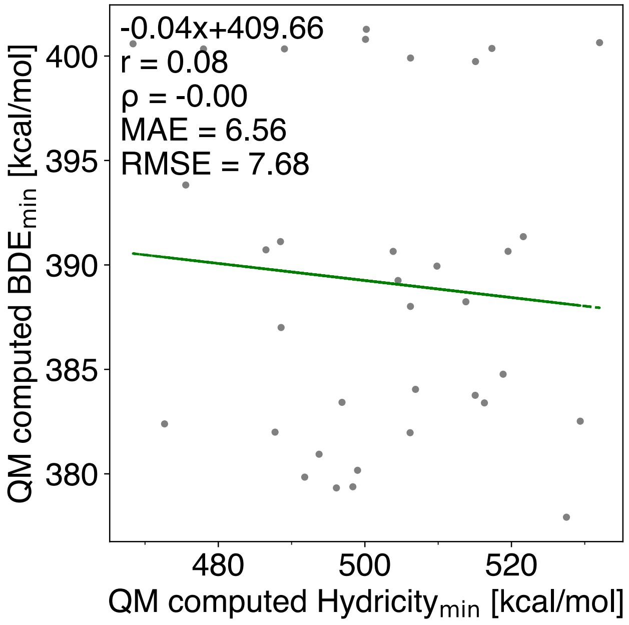

Figure S16: Correlating QM computed hydricities (ΔG°_{min}) and QM computed BDE (ΔG°_{min}) values for 35 compounds[10]. r: Pearson correlation coefficient; ρ: Spearman's rank correlation coefficient; MAE: mean absolute error; RMSE: root mean squared error. QM calculations are carried out at the M06-2X/def2-TZVP CPCM(DMSO)//GFN2-xTB ALPB(DMSO) level of theory.

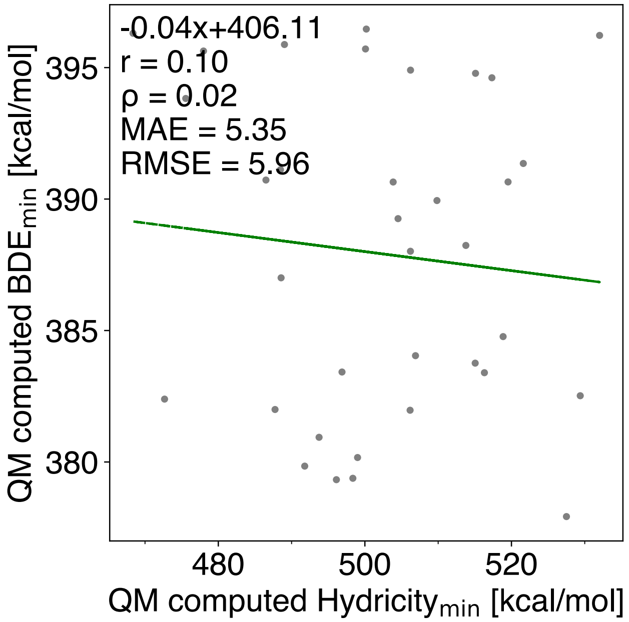

Figure S17: Correlating QM computed hydricities (ΔG°_{min}) and QM computed BDE (ΔG°_{min}) values for 35 compounds[10]. r: Pearson correlation coefficient; ρ: Spearman's rank correlation coefficient; MAE: mean absolute error; RMSE: root mean squared error. QM calculations are carried out at the r^{2}SCAN-3c/def2-mTZVPP/J CPCM(DMSO)//GFN2-xTB ALPB(DMSO) level of theory.

4. The descriptor

The following section evaluates the correlation between ML predicted hydricities and QM computed hydricities for each descriptor. From S3, it is seen that the charge shell descriptor with six shells and values sorted according to the Cahn—Ingold—Prelog (CIP) rules best describes the hydricities when looking at MAE and RMSE values. However, we have chosen to proceed with the descriptors with the three numbers of shells. The reason is that the dimensions are up to a factor of 27 higher for six shells. We also expect a better overall performance as three shells better describe the local environment for each atom. We also note that the maximum absolute error is lower for the three-shell descriptor, which may improve the overall performance.

Table S3: Performance metrics using different numbers of shells (n-shells) for the descriptor vector for the QM dataset (740 compounds; 3278 hydricities). MAE: mean absolute error; RMSE: root mean squared error. LightGBM regressor with default parameters

Descriptor	n-shells	Dimensions	r	ρ	MAE	RMSE	MaxAE
Sorted-shell	3	53	0.98	0.97	2.30	3.74	23.1
Sorted-shell	4	161	0.98	0.97	2.23	3.68	22.9
Sorted-shell	5	485	0.98	0.97	2.23	3.69	23.9
Sorted-shell	6	1457	0.98	0.98	2.19	3.65	23.6

5. Machine learning models

5.1 Test set

In a manner analogous to ref[19], regression and binary classification models have been trained to evaluate how well we can predict hydricity for each site or reaction site (compared to QM). S4 shows the performance metrics for the different regression models using 5-fold cross-validation, where the correlation between ML predicted hydricities and QM computed hydricities are shown in S18.

Based on the classification of the minimum hydricity, each site in a molecule is set to either a '1' (lowest hydricity site) or '0' (not lowest hydricity site. We also introduce a tolerance where a hydricity value within +1 kcal/mol or +2 kcal/mol of the lowest hydricity is accepted as '1' to account for slight variations. From that, a confusion matrix (CM) is constructed to compare the ML predicted sites to the QM computed sites. If the ML model's prediction aligns with the QM computed sites, a site is classified as a true positive (TP) or true negative (TN). Conversely, it is labeled as a false positive (FP) or false negative (FN) if the ML predicted site differs from the QM computed site. From these classifications, we derive several evaluation parameters: Accuracy (ACC), Matthew's correlation coefficient (MCC), recall/sensitivity (true-positive rate - TPR), specificity (true-negative rate - TNR), precision (positive predictive value - PPV), and negative predictive value (NPV). A summary of the best ML models for predicting the reaction site is found inS5. For more details, see S6 for the classifiers and S7 for a comprehensive comparison between the different models for the test set.

Table S4: Performance metrics for different regression models: The QM dataset (740 compounds; 3278 hydricities) is randomly split into a training set (80 %; 595 compounds; 2607 hydricities) and a held-out test set (20 %; 145 compounds; 671 hydricities). Subsequently, a 5-fold random shuffled cross-validation (CV) is conducted. Within each fold, the original training set is split randomly into a new training set (90 % of the original training set) and a validation set (10 % of the original training set). μ MAE: mean mean absolute error for 5-fold CV; μ RMSE: mean root mean squared error for 5-fold CV; MAE: mean absolute error; RMSE: root mean squared error. The best model is marked in bold.

Method	Train		Valid		Test	
LightGBM regressor n-shells 3						
Regression	μ MAE	μ RMSE	μ MAE	μ RMSE	MAE	RMSE
**Regressor DART (default)**	$0.17 \pm 7.7 \cdot 10^{-3}$	0.40 ± 1.8	3.03 ± 0.32	5.21 ± 1.10	**2.30**	**3.74**
Regressor DART	$0.35 \pm 1.60 \cdot 10^{-2}$	0.62 ± 0.11	2.96 ± 0.28	5.13 ± 1.14	2.33	3.84
Regressor GBDT (default)	0.68 ± 0.32	1.18 ± 0.42	3.0 ± 0.36	5.17 ± 1.01	2.45	3.92
Regressor GBDT	1.41 ± 0.64	2.22 ± 1.03	3.12 ± 0.35	5.37 ± 1.05	2.47	3.96

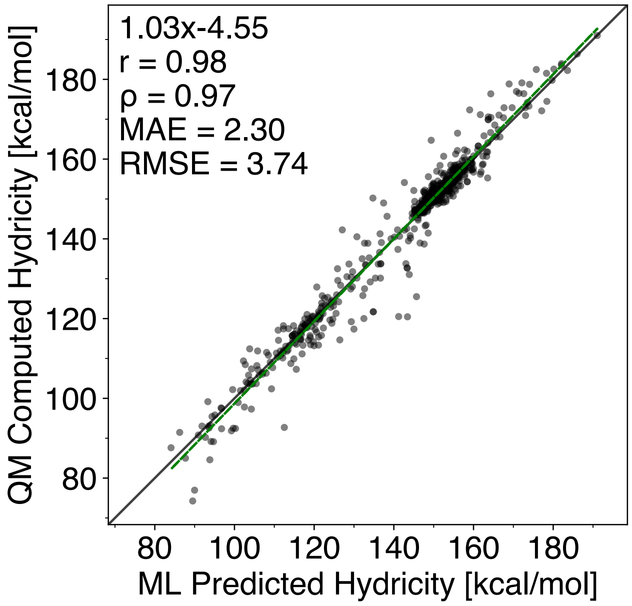

Figure S18: ML predicted hydricities vs. QM computed hydricities of the held-out test set (145 compounds; 671 hydricities). r: Pearson correlation coefficient; ρ: Spearman's rank correlation coefficient; MAE: mean absolute error; RMSE: root mean squared error. All predictions are carried out using the best ligthGBM regressor. All calculations are carried out at the r^{2}SCAN-3c/def2-TZVPPD CPCM(DMSO)//GFN2-xTB ALPB(DMSO) level of theory.

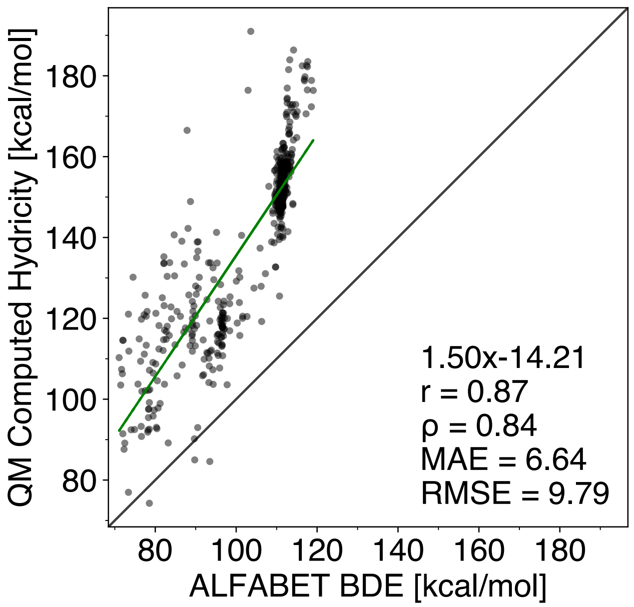

Figure S19: ALFABET BDEs vs. QM computed hydricities of the held-out test set (145 compounds; 671 hydricities). r: Pearson correlation coefficient; ρ: Spearman's rank correlation coefficient; MAE: mean absolute error; RMSE: root mean squared error. All predictions are carried out using the best ligthGBM regressor. All calculations are carried out at the r^{2}SCAN-3c/def2-TZVPPD CPCM(DMSO)//GFN2-xTB ALPB(DMSO) level of theory.

Table S5: Held-out test set (145 compounds; 671 hydricities) performance metrics for binary classification with the best regression model (Regressor DART (default) and the best classification model (Classifier GBDT (default). Each hydricity in a molecule is set to either a '1' (lowest hydricity site) or '0' (not lowest hydricity site. For the null model, all sites are set to '0'. The best models are marked in bold. ACC: Accuracy; MCC: Matthew's correlation coefficient; PPV: precision/positive predictive value; TPR: recall/true-positive rate; TNR: specificity/true-negative rate; NPV: negative predictive value.

Method	ACC	MCC	PPV	TPR	TNR	NPV
Zero rate null model	0.78	0.00	0.00	0.00	1.00	0.78
Random Classifier	0.68	0.11	0.32	0.31	0.79	0.79
**Regressor**	0.96	0.88	0.90	0.91	0.97	0.98
Classifier	0.90	0.69	0.79	0.71	0.95	0.92
ALFABET	0.89	0.67	0.82	0.66	0.96	0.91

Table S6: Performance metrics for different regression models: The QM dataset (740 compounds; 3278 hydricities) is randomly split into a training set (80 %; 595 compounds; 2607 hydricities) and a held-out test set (20 %; 145 compounds; 671 hydricities). Subsequently, a 5-fold random shuffled cross-validation (CV) is conducted. Within each fold, the original training set is split randomly into a new training set (90 % of the original training set) and a validation set (10 % of the original training set). μ AUC: Area under the curve for 5-fold CV; μ logloss: mean logloss for 5-fold CV.

Method	training set		validation set	
	μ AUC	μ logloss	μ AUC	μ logloss
LightGBM classifier n-shells 3				
Classifier DART (default)	1.00 ± 0.00	0.00 ± 0.00	0.94 ± 0.02	0.77 ± 0.23
Classifier+1 DART (default)	1.00 ± 0.00	0.00 ± 0.00	0.93 ± 0.03	0.82 ± 0.33
Classifier+2 DART (default)	1.00 ± 0.00	0.00 ± 0.00	0.92 ± 0.03	0.92 ± 0.36
	-	-	-	-
Classifier DART	1.00 ± 0.00	0.04 ± 0.00	0.94 ± 0.02	0.29 ± 0.07
Classifier+1 DART	1.00 ± 0.00	0.06 ± 0.00	0.93 ± 0.03	0.31 ± 0.08
Classifier+2 DART	1.00 ± 0.00	0.07 ± 0.00	0.92 ± 0.03	0.34 ± 0.09
	-	-	-	-
Classifier GBDT (default)	1.00 ± 0.00	0.09 ± 0.03	0.94 ± 0.02	0.27 ± 0.05
Classifier+1 GBDT (default)	0.99 ± 0.01	0.13 ± 0.06	0.94 ± 0.02	0.29 ± 0.07
Classifier+2 GBDT (default)	0.99 ± 0.01	0.12 ± 0.03	0.93 ± 0.03	0.31 ± 0.07
	-	-	-	-
Classifier GBDT	1.00 ± 0.00	0.10 ± 0.05	0.95 ± 0.02	0.26 ± 0.07
Classifier+1 GBDT	0.99 ± 0.01	0.13 ± 0.05	0.93 ± 0.03	0.28 ± 0.08
Classifier+2 GBDT	1.00 ± 0.00	0.11 ± 0.04	0.93 ± 0.03	0.30 ± 0.07

Table S7: Held-out test set (145 compounds; 671 hydricities) performance metrics for different binary classification models, including the regression models. The QM dataset (740 compounds; 3278 hydricities) is randomly split by compound into a training set (80 %; 595 compounds; 2607 hydricities) and a held-out test set (20 %; 145 compounds; 671 hydricities). Each hydricity in a molecule is set to either a '1' (lowest hydricity site) or '0' (not lowest hydricity site. +1 and +2 denote either +1 kcal/mol or +2 kcal/mol of the lowest hydricity value accepted as '1' (true site). For the null model, all sites are set to '0'. The best models are marked in bold. ACC: Accuracy; MCC: Matthew's correlation coefficient; PPV: precision/positive predictive value; TPR: recall/true-positive rate; TNR: specificity/true-negative rate; NPV: negative predictive value.

Method	ACC	MCC	PPV	TPR	TNR	NPV
Zero rate null model	0.78	-	-	-	-	-
Random classifer	0.68	0.11	0.32	0.31	0.79	0.79
LightGBM regressor n-shells 3						
**Regressor DART (default)**	0.96	0.88	0.90	0.91	0.97	0.98
Regressor+1 DART (default)	0.95	0.86	0.90	0.89	0.97	0.97
Regressor+2 DART (default)	0.94	0.85	0.90	0.87	0.97	0.95
	-	-	-	-	-	-
Regressor DART	0.94	0.81	0.85	0.86	0.96	0.96
Regressor+1 DART	0.94	0.83	0.87	0.86	0.96	0.96
Regressor+2 DART	0.92	0.80	0.87	0.83	0.96	0.94
	-	-	-	-	-	-
Regressor GBDT (default)	0.95	0.86	0.88	0.89	0.97	0.97
Regressor+1 GBDT (default)	0.95	0.86	0.90	0.88	0.97	0.96
Regressor+2 GBDT (default)	0.93	0.83	0.89	0.85	0.96	0.95
	-	-	-	-	-	-
Regressor GBDT	0.95	0.86	0.89	0.90	0.97	0.97
Regressor+1 GBDT	0.95	0.85	0.90	0.87	0.97	0.96
Regressor+2 GBDT	0.93	0.82	0.88	0.85	0.96	0.95
LightGBM classifier n-shells 3						
Classifier DART (default)	0.89	0.67	0.76	0.71	0.94	0.92
Classifier+1 DART (default)	0.88	0.65	0.73	0.71	0.93	0.92
Classifier+2 DART (default)	0.87	0.64	0.71	0.73	0.92	0.92
	-	-	-	-	-	-
Classifier DART	0.87	0.63	0.71	0.71	0.92	0.92
Classifier+1 DART	0.87	0.63	0.68	0.75	0.90	0.93
Classifier+2 DART	0.87	0.64	0.67	0.79	0.89	0.94
	-	-	-	-	-	-
**Classifier GBDT (default)**	0.90	0.69	0.79	0.71	0.95	0.92
Classifier+1 GBDT (default)	0.88	0.65	0.75	0.70	0.94	0.92
Classifier+2 GBDT (default)	0.89	0.68	0.74	0.75	0.93	0.93
	-	-	-	-	-	-
Classifier GBDT	0.89	0.67	0.77	0.70	0.94	0.92
Classifier+1 GBDT	0.88	0.64	0.75	0.68	0.94	0.91
Classifier+2 GBDT	0.88	0.64	0.71	0.73	0.92	0.93
ALFABET	0.89	0.67	0.82	0.66	0.96	0.91
ALFABET+1	0.89	0.68	0.85	0.65	0.96	0.90
ALFABET+2	0.88	0.66	0.86	0.62	0.97	0.88

Table S8: Held-out test set (145 compounds; 671 hydricities) performance metrics for different binary classification models, including the best regression model. The QM dataset (740 compounds; 3278 hydricities) is randomly split by compound into a training set (80 %; 595 compounds; 2607 hydricities) and a held-out test set (20 %; 145 compounds; 671 hydricities). Each hydricity in a molecule is set to either a '1' (lowest hydricity site) or '0' (not lowest hydricity site. +1 and +2 denote either +1 kcal/mol or +2 kcal/mol of the lowest hydricity value accepted as '1' (true site). For the null model, all sites are set to '0'. The best models are marked in bold. TP: True positives; TN: True negatives; FP: False positives; FN: False negatives.

Method	TP	TN	FP	FN
Null model	0	525	0	146
Random classifier	23	193	50	51
LightGBM Regressor n-shells 3				
**Regressor DART (default)**	133	511	14	13
Regressor+1 DART (default)	141	497	15	18
Regressor+2 DART (default)	151	481	16	23
Classifier n-shells 3				
Classifier DART (default)	101	493	32	45
Classifier+1 DART (default)	103	493	32	43
Classifier+2 DART (default)	109	487	38	37
	-	-	-	-
Classifier DART	113	488	37	33
Classifier+1 DART	115	485	40	31
Classifier+2 DART	116	476	49	30
	-	-	-	-
**Classifier GBDT (default)**	104	499	26	42
Classifier+1 GBDT (default)	105	495	30	41
Classifier+2 GBDT (default)	107	488	37	39
	-	-	-	-
Classifier GBDT	104	495	30	42
Classifier+1 GBDT	105	494	31	41
Classifier+2 GBDT	110	486	39	36
ALFABET	96	504	21	50
ALFABET+1	104	493	19	55
ALFABET+2	108	480	17	66

5.2 Outliers for the test set

This section examines the outliers associated with the held-out test set (20 %; 145 compounds; 671 hydricities) for the best regression model, see see Figure S20 and Table S9. The outliers were checked to see if the QM calculations failed or if the conformations were wrong. However, this was not the case. For compounds ibond193, comp294, shen50, and ibond301, the high difference in hydricities between QM and ML occurs at the allyl-like cations. The ML model consequently predicts these cations to be less stable than the QM method. This is likely due to the extra stability gained through resonance effects that the ML model cannot describe. Resonance or inductive effects from electron-donating- or electron-withdrawing groups (EDG, EWG) seem to be a factor for the outliers. Another example is for comp227, where the ML model predicts the lowest hydricity site to be 8 (QM: 109.39; ML 102.26), but the QM calculations predict the lowest hydricity site to be 0 (QM: 92.71; ML 112.51). The carbocation at site 8 has an antiaromatic character and is thus destabilized. Our model is likely to have difficulty capturing the extra stabilization due to conjugation, because the CM5 atomic charge descriptor vector is derived from the neutral molecule. A more precise way to construct this descriptor vector would be to either concatenate the CM5 charges corresponding to each protonated site in the molecule, or to use the difference between the neutral and protonated descriptor vectors for each site. Either of these strategies would yield a more faithful representation of the molecule. It is most likely that the training set (80 %; 595 compounds; 2607 hydricities) contains no to few compounds or types of sites that we see in the test set. Thus, the ML model has difficulty distinguishing the different sites where, e.g., the difference between an EWG or EDG in the vicinity significantly impacts the carbocation stability. Therefore, a more extensive training set for our ML model would be advantageous.

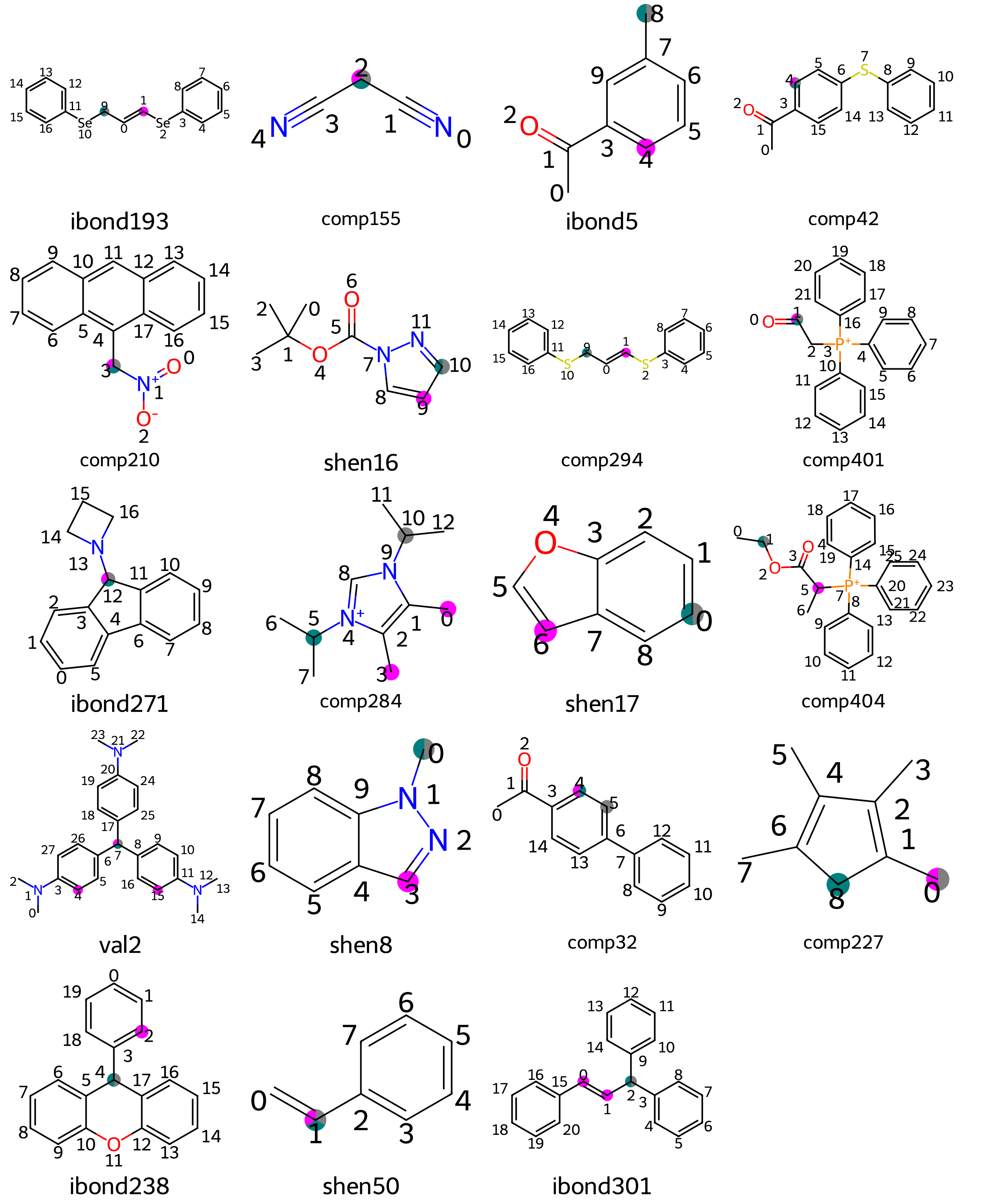

Figure S20: Outliers (19 compounds, 23 outliers) from the held-out test set (145 compounds; 671 hydricities) using the best regression model. The outliers have an error of 10 or above between the QM computed hydricity and the ML predicted hydricity (magenta); Lowest QM site (grey); lowest ML site (teal).

Table S9: Outliers (19 compounds, 23 outliers) from the held-out test set (145 compounds; 671 hydricities) using the best regression model. The outliers below have an error of 10 or above between the QM computed hydricity and the ML predicted hydricity. * denotes the lowest QM computed site.

names	atom index	$\text{Hydricity}_{\text{exp}}$	$\text{Hydricity}_{\text{QM}}$	$\text{Hydricity}_{\text{ML}}$	error QM vs ML
ibond193	1	-	119.22	129.32	10.09
comp155	1*	-	166.50	155.99	10.51
ibond5	4	-	154.12	142.65	11.47
comp42	4*	-	149.00	137.43	11.57
comp210	3*	-	114.66	126.44	11.79
shen16	9	-	176.37	164.33	12.04
comp294	1	-	120.017	132.48	12.47
comp401	1	-	117.36	129.99	12.62
ibond271	12*	-	76.97	89.99	13.02
comp284	0	-	121.71	134.84	13.14
comp284	3	-	121.71	134.84	13.14
shen17	6	-	172.34	159.03	13.31
comp404	5	-	142.25	127.06	15.19
val2	4	76.0	132.71	143.35	10.64
val2	7*	76.0	74.26	89.51	15.25
val2	15	76.0	132.69	143.35	10.66
shen8	3	-	164.72	149.37	15.34
comp32	4	-	150.22	134.75	15.48
comp227	0	-	92.71	112.51	19.81
ibond238	2	-	125.51	145.69	20.19
shen50	1	-	120.52	141.21	20.69
ibond301	0	-	120.45	143.43	22.97
ibond301	1	-	131.05	143.88	12.82

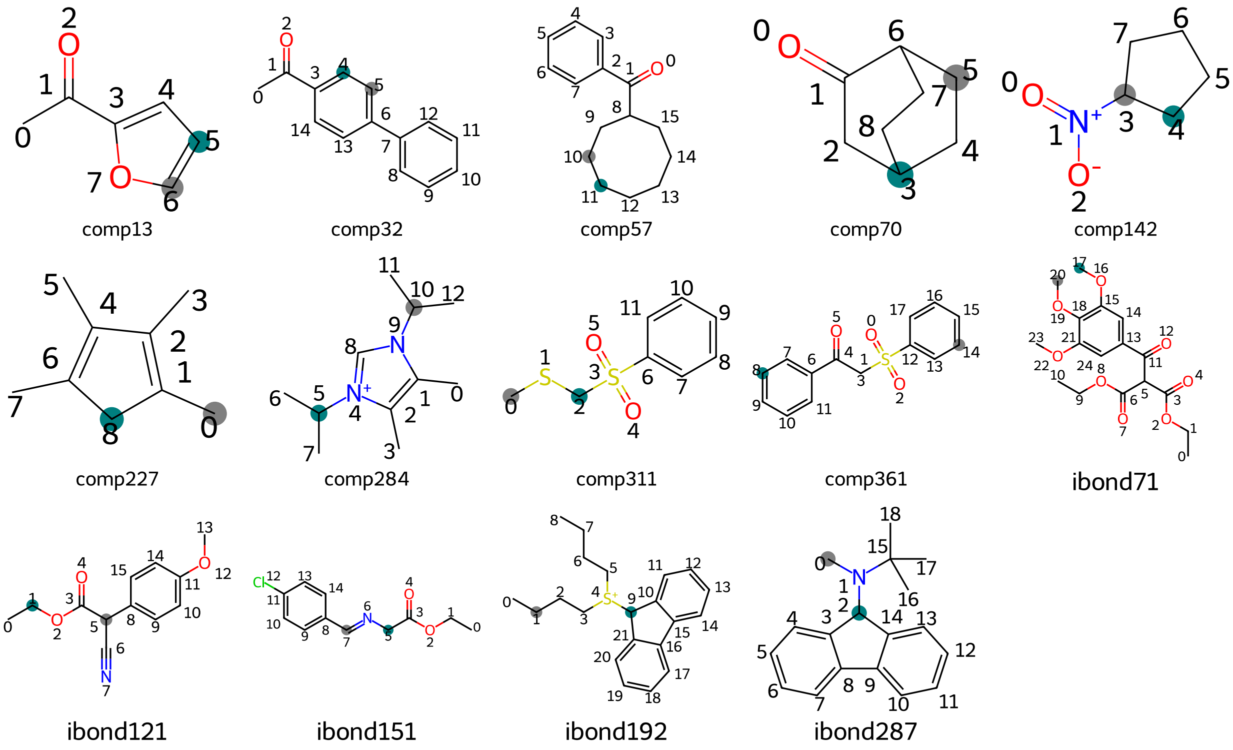

Figure S21: False negatives (FN) and false positives (FP) from the held-out test set (145 compounds; 671 hydricities) using the best regression model as a binary classifier. Lowest QM site (grey); lowest ML site (teal).

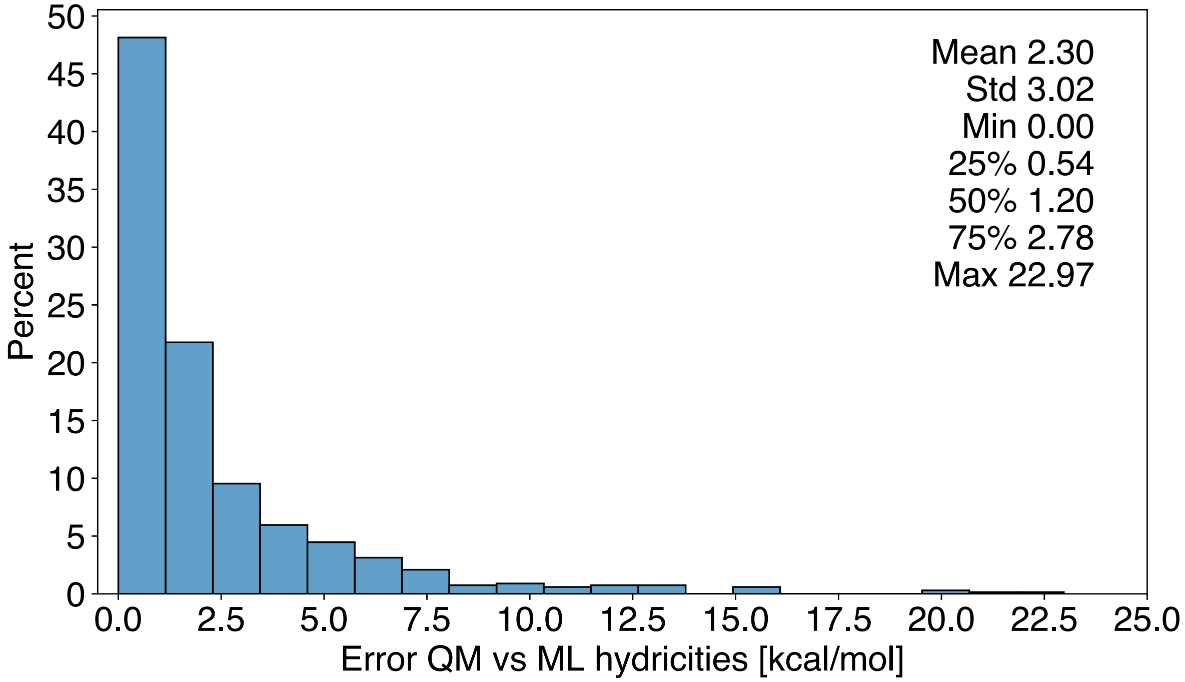

Figure S22: Histogram of the percentage of the hydricities within a given error between QM and ML from the held-out test set (145 compounds; 671 hydricities) using the best regression model.

References

(1) Neese, F. The ORCA program system. *WIREs Computational Molecular Science* **2012**, *2*, 73–78.

(2) Neese, F.; Wennmohs, F.; Becker, U.; Riplinger, C. The ORCA quantum chemistry program package. *The Journal of Chemical Physics* **2020**, *152*, 224108.

(3) Grimme, S.; Hansen, A.; Ehlert, S.; Mewes, J.-M. r ^{2} SCAN-3c: A "Swiss army knife" composite electronic-structure method. *The Journal of Chemical Physics* **2021**, *154*, 064103.

(4) Zhao, Y.; Truhlar, D. G. The M06 suite of density functionals for main group thermochemistry, thermochemical kinetics, noncovalent interactions, excited states, and transition elements: two new functionals and systematic testing of four M06-class functionals and 12 other functionals. *Theoretical Chemistry Accounts* **2008**, *120*, 215–241.

(5) Weigend, F.; Ahlrichs, R. Balanced basis sets of split valence, triple zeta valence and quadruple zeta valence quality for H to Rn: Design and assessment of accuracy. *Physical Chemistry Chemical Physics* **2005**, *7*, 3297.

(6) Rappoport, D.; Furche, F. Property-optimized Gaussian basis sets for molecular response calculations. *The Journal of Chemical Physics* **2010**, *133*, 134105.

(7) Yanai, T.; Tew, D. P.; Handy, N. C. A new hybrid exchange–correlation functional using the Coulomb-attenuating method (CAM-B3LYP). *Chemical Physics Letters* **2004**, *393*, 51–57.

(8) Caldeweyher, E.; Ehlert, S.; Hansen, A.; Neugebauer, H.; Spicher, S.; Bannwarth, C.; Grimme, S. A generally applicable atomic-charge dependent London dispersion correction. *The Journal of Chemical Physics* **2019**, *150*, 154122.

(9) Barone, V.; Cossi, M. Quantum Calculation of Molecular Energies and Energy Gradients in Solution by a Conductor Solvent Model. *The Journal of Physical Chemistry A* **1998**, *102*, 1995–2001.

(10) Cheng, J.; Handoo, K. L.; Parker, V. D. Hydride affinities of carbenium ions in acetonitrile and dimethyl sulfoxide solution. *Journal of the American Chemical Society* **1993**, *115*, 2655–2660, Publisher: American Chemical Society.

(11) Ellis, W. W.; Raebiger, J. W.; Curtis, C. J.; Bruno, J. W.; DuBois, D. L. Hydricities of BzNADH, $C_5H_5Mo(PMe_3)(CO)_2H$, and $C_5Me_5Mo(PMe_3)(CO)_2H$ in Acetonitrile. *Journal of the American Chemical Society* **2004**, *126*, 2738–2743.

(12) Handoo, K. L.; Cheng, J. P.; Parker, V. D. Hydride affinities of organic radicals in solution. A comparison of free radicals and carbenium ions as hydride ion acceptors. *Journal of the American Chemical Society* **1993**, *115*, 5067–5072.

(13) Cheng, J.-P.; Lu, Y.; Zhu, X.; Mu, L. Energetics of Multistep versus One-step Hydride Transfer Reactions of Reduced Nicotinamide Adenine Dinucleotide (NADH) Models with Organic Cations and *p* -Quinones. *The Journal of Organic Chemistry* **1998**, *63*, 6108–6114.

(14) Tsay, C.; Livesay, B. N.; Ruelas, S.; Yang, J. Y. Solvation Effects on Transition Metal Hydricity. *Journal of the American Chemical Society* **2015**, *137*, 14114–14121.

(15) Brereton, K. R.; Smith, N. E.; Hazari, N.; Miller, A. J. M. Thermodynamic and kinetic hydricity of transition metal hydrides. *Chemical Society Reviews* **2020**, *49*, 7929–7948.

(16) St. John, P. C.; Guan, Y.; Kim, Y.; Etz, B. D.; Kim, S.; Paton, R. S. Quantum chemical calculations for over 200,000 organic radical species and 40,000 associated closed-shell molecules. *Scientific Data* **2020**, *7*, 244, Publisher: Nature Publishing Group.

(17) St. John, P. C.; Guan, Y.; Kim, Y.; Kim, S.; Paton, R. S. Prediction of organic homolytic bond dissociation enthalpies at near chemical accuracy with sub-second computational cost. *Nature Communications* **2020**, *11*, 2328.

(18) V, S. S. S.; Kim, Y.; Kim, S.; John, P. C. S.; Paton, R. S. Expansion of bond dissociation prediction with machine learning to medicinally and environmentally relevant chemical space. *Digital Discovery* **2023**, *2*, 1900–1910, Publisher: RSC.

(19) Borup, R. M.; Ree, N.; Jensen, J. H. pKalculator: A pKa predictor for C–H bonds. *Beilstein Journal of Organic Chemistry* **2024**, *20*, 1614–1622, Publisher: Beilstein-Institut.
